# T-KDE: a method for genome-wide identification of constitutive protein binding sites from multiple ChIP-seq data sets

**DOI:** 10.1186/1471-2164-15-27

**Published:** 2014-01-15

**Authors:** Yuanyuan Li, David M Umbach, Leping Li

**Affiliations:** 1 Biostatistics Branch, National Institute of Environmental Health Sciences, Research Triangle Park, Morrisville, NC 27709, USA

**Keywords:** Binding pattern, ChIP-seq, Kernel density estimation, Binary range tree, Mode-finding, Constitutive site, CTCF code

## Abstract

**Background:**

A protein may bind to its target DNA sites constitutively, i.e., regardless of cell type. Intuitively, constitutive binding sites should be biologically functional. A prerequisite for understanding their functional relevance is knowing all their locations for a protein of interest. Genome-wide discovery of constitutive binding sites requires robust and efficient computational methods to integrate results from numerous binding experiments. Such methods are lacking, however.

**Results:**

To locate constitutive binding sites for a protein using ChIP-seq data for that protein from multiple cell lines, we developed a method, T-KDE, which combines a binary range tree with a kernel density estimator. Using 132 CTCF (CCCTC-binding factor) ChIP-seq datasets, we showed that the number of constitutive sites identified by T-KDE is robust to the choice of tuning parameter and that T-KDE identifies binding site locations more accurately than a binning approach. Furthermore, T-KDE can identify constitutive sites that are missed by a motif-based approach either because a bound site failed to reach the motif significance cutoff or because the peak sequence scanned was too short. By studying sites declared constitutive by T-KDE but not by the motif-based approach, we discovered two new CTCF motif variants. Using ENCODE data on 22 transcription factors (TF) in 132 cell lines, we identified constitutive binding sites for each TF and provide evidence that, for some TFs, they may be biologically meaningful.

**Conclusions:**

T-KDE is an efficient and effective method to predict constitutive protein binding sites using ChIP-seq peaks from multiple cell lines. Besides constitutive binding sites for a given protein, T-KDE can identify genomic “hot spots” where several different proteins bind and, conversely, cell-type-specific sites bound by a given protein.

## Background

**Figure 1:**
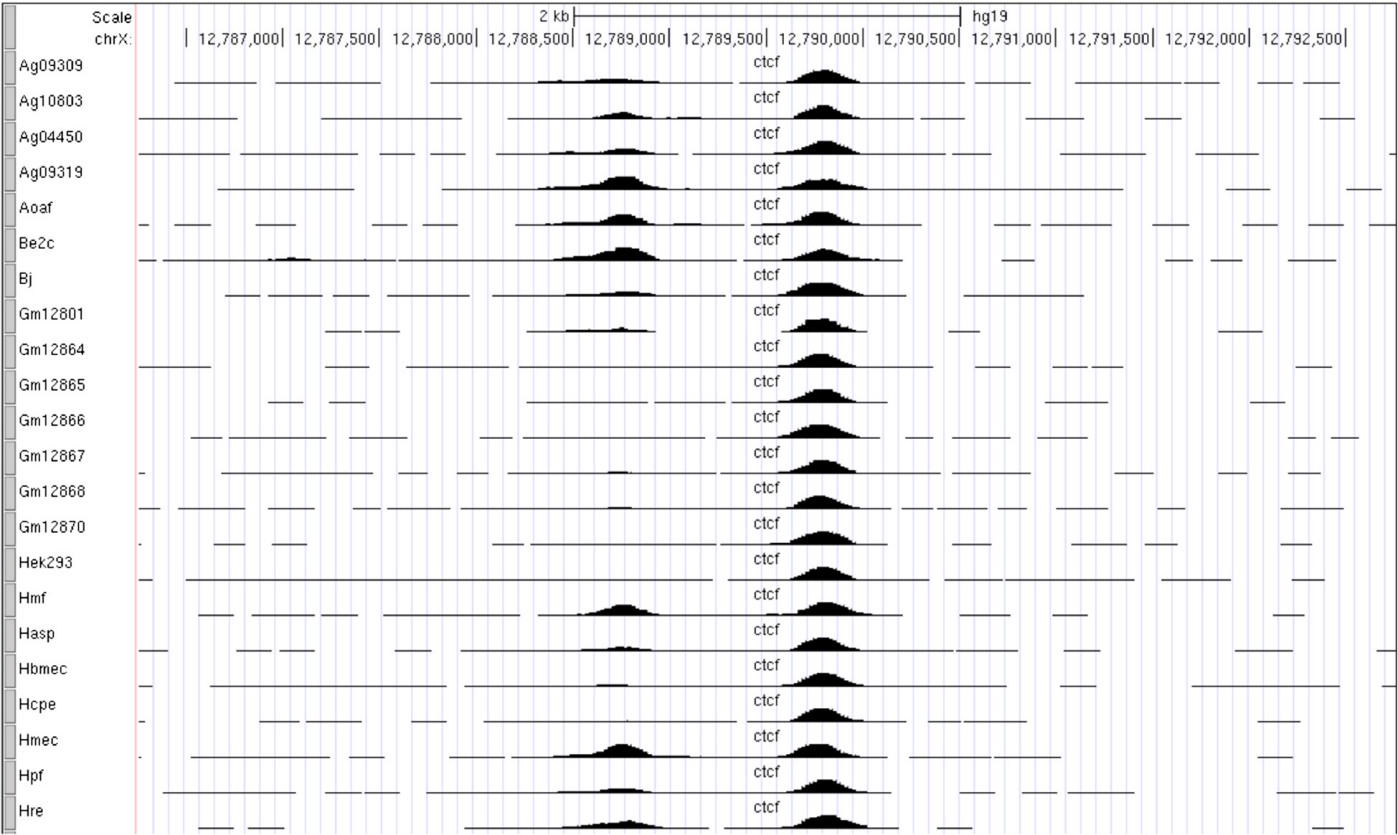
**An example of one constitutive and one non-constitutive CTCF binding site in a restricted region on chromosome X.** Each track (for the cell line indicated on the left) displays the CTCF binding profile in the region using UCSC big wiggle format. The locus to the right of center where a CTCF binding peak appears in all cell lines displayed would be declared constitutive whereas the locus to the left of center where a binding peak is present less often would be non-constitutive.

Transcription factors (TFs) are important components of gene transcriptional regulation. The binding of a TF to a specific locus can be developmental-stage or cell-type specific; alternatively, as growing evidence suggests, sometimes a protein binds to a specific locus constitutively, i.e., in all the cell types studied so far. A good example is the CCCTC-binding factor (CTCF). Studies using chromatin immunoprecipitation followed by microarray (ChIP-chip) or sequencing (ChIP-seq) showed that, unlike many other TFs/proteins, a portion of the CTCF sites are constitutively bound [1,2] as illustrated in Figure 1. We believe that these constitutive binding sites are likely to have unique or fundamental biological roles. Recently, we carried out a comprehensive analysis of the 116 CTCF ChIP-seq datasets from 56 cell lines from the ENCODE (Encyclopedia of DNA Elements) consortium and identified ~24,000 CTCF binding sites that were bound in more than 90% of the 56 cell lines [3] . Because these constitutive CTCF binding sites were enriched among CTCF-mediated long-range chromatin interactions in K562 and MCF7 cell lines, we hypothesized that these constitutive CTCF binding sites might play a role in maintaining and/or establishing chromatin structures common to all cell types [4]. Thus, we see value in locating constitutive binding sites for other DNA binding proteins as a possible window into highly conserved biological processes.

Many TFs bind to DNA directly and have well-defined motif models. For such TFs, binding sites may be located by scanning their ChIP-seq peak sequences with motif models like position weight matrices (PWM) [5]. Using ChIP-seq data for the same TF from a number of cell lines, one would consider a binding site constitutive if it were found in a sufficiently high proportion of the available cell lines/types. We refer to this as the motif-based approach. Not every transcription factor has a known binding motif, however. Among the ~1,400 sequence-specific DNA-binding transcription factors in the human genome [6], only about 10-20% of them have known binding motifs [7]. Thus, while the motif-based approach should work well for factors with well-defined PWMs such as CTCF, it will fail for TFs lacking reliable PWMs or for proteins that do not always bind to their target DNA sites directly.

A simple alternative approach, one that accommodates TFs that binds indirectly or that lack a well-defined PWM, is to divide the genome into fixed width bins and count the number of peak centers from ChIP-seq that fall into each bin, e.g., [8] and [9]. Bins containing peak centers for a sufficiently high proportion of the available cell lines/types are declared as constitutive. Although this binning method is simple, intuitive and commonly used in genome analysis, it suffers from several drawbacks, including a boundary effect where which of two adjacent bins contains a peak center may be ambiguous.

Our T-KDE approach is based on the following idea. If a particular genomic locus is bound by a protein in all available cell lines, then the centers of all ChIP-seq peaks, one from each cell line, at that locus should be within close proximity (as in Figure 1). We aimed to systematically identify such sites from ChIP-seq experiments that target the same protein in multiple cell lines by simultaneously analyzing peak centers across all the experiments.

Our goal is distinct from peak calling in a single ChIP-seq experiment. A ChIP-seq peak is a genomic region (~100 to 500 bps for a typical transcription factor) enriched with sequence reads and identified using a peak-calling algorithm, e.g. [10,11]. Various peak calling algorithms find genomic regions enriched for a binding signal in a variety of data types. These include Hidden Markov Model (HMM)-based peak calling algorithms for ChIP-seq data [11], for ChIP-chip data [12,13], and for MeDIP-seq data [14]. All identify peaks by modeling emission and transition probabilities using multiple states and exploiting distinct signal signatures in different states.

Despite the distinct goals, another approach for detecting constitutive protein binding sites might be to apply existing peak-calling tools to the original ChIP-seq reads from multiple cell lines simultaneously, expecting that constitutive binding sites will exhibit especially high peaks. Such an approach has several drawbacks. First, BAM files from individual ChIP-seq experiments can be very large, so that combining and processing BAM files from tens or hundreds of experiments together will be computationally intensive. Secondly, combining read counts from multiple data sets where some binding occurs at loci common across many data sets and some binding occurs at loci specific to particular data sets will introduce unusual patterns variation in reads counts that could bias estimation of background rates. For tools that require estimation of background models, this feature may compromise their ability to reliably detect constitutive binding sites. Finally, the definition of constitutive binding site in terms of binding in most cell lines to the same site does not directly translate to a criterion based on peak height in a combined BAM file. Consequently, declaring a constitutive peak seems to require mapping all reads under each detected peak back to their original BAM files – an additional computational burden.

In this paper, we propose an effective and efficient alternative to binning for locating binding sites for TFs that may bind directly or indirectly. Like binning, it uses peak centers from ChIP-seq as input data. Our algorithm, T-KDE, identifies binding site locations by combining a kernel density estimator (KDE) with a binary range tree. Kernel density estimation, also known as the Parzen window method, is an unsupervised and non-parametric technique for estimating a continuous probability density function from sample data [15,16]. Because KDEs can converge asymptotically to any density function [16], they are widely used and have been applied to many genomic problems such as ChIP-seq peak calling [17], analyzing nucleosome positioning [18] and detecting transcription factor binding motifs based on their over-representation in regulatory regions [19]. In this paper, we use a KDE to find those genomic regions that contain the highest density of ChIP-seq peak centers from multiple cell lines/types for a given TF. Use of a binary range tree in conjunction with kernel density estimation enhances T-KDE’s speed. A binary range tree is a helpful algorithm for many applications involving range or nearest neighbor searches, indexing and clustering [20-22]; we use it to recursively subdivide the set of peak centers into subgroups that allow efficient density estimation and mode finding.

Using information on the location of peak centers from 132 CTCF ChIP-seq datasets from the ENCODE project, we compared T-KDE to both the motif-based approach and the binning approach. T-KDE outperformed the binning approach and was competitive with the motif-based approach. More than 90% of the T-KDE-declared constitutive CTCF binding sites were within 20 base pairs (bp) from the nearest motif-declared constitutive CTCF binding sites (16-bp canonical motif) — indicating that T-KDE is highly accurate. In addition, T-KDE also identified additional constitutive CTCF binding sites that the motif-based approach failed to find due to lack of apparent motif sites in the ChIP-seq peaks. We also applied T-KDE to 21 other proteins for which replicate ChIP-seq datasets were available in six or more cell lines and found that the number of constitutive binding sites varied from less than a hundred to tens of thousands. Gene ontology (GO) analysis of the genes with constitutive binding sites in their promoters suggests that constitutive binding sites for several of the proteins are biologically meaningful.

## Methods

### Data

We downloaded data on ChIP-seq peaks for 22 transcription factors (in Additional file 1: Table S1) from the ENCODE portal at the UCSC Genome Browser [23]. (The complete list of datasets and their unique identifiers can be found in Additional file 1: Table S2.) For each ChIP-seq peak, we calculated the location of the peak center as half the sum of the start and end coordinates for the peak, and we used these locations for subsequent analysis.

### Location of constitutive CTCF binding sites via motif model: our “gold standard”

For each of 132 CTCF ChIP-seq datasets with at least one replicate, we extended/trimmed each peak to 200 bp in length from its center. We then used a custom Python code to extract the sequences from the GRCh37 assembly stored locally. Next, we predicted the locations of the CTCF binding sites in the sequences using the GADEM software [24] with a CTCF position weight matrix (PWM) derived previously [24] (see in Additional file 1: Table S3). We declared a subsequence a CTCF binding site when its PWM score exceeded the score corresponding to the *p*-value cutoff of 0.0005. When more than one CTCF site was found in the sequence for a single peak, only the highest scoring site (with the lowest *p*-value) was retained for that peak. When a CTCF binding site was found in two or more replicate datasets representing a single cell line, the site was declared present in that cell line. A CTCF binding site was considered a constitutive binding site when the same motif site was present in more than 90% of the 55 cell lines. We used the center of the motif site as the location of the motif-based binding site.

### Identification of constitutive binding sites via binning

We divided each chromosome of the human genome into bins of equal size beginning at the centromere and proceeding outward along each arm (the final bin on each arm might be smaller than the others). The center of any bin containing peak centers from at least 2 replicate datasets from the same cell line was declared a binding site location as before, and those bins containing peak centers for more than 90% of the cell lines were declared constitutive. We examined this binning procedure with various bin sizes ranging from 100 to 1000 bp.

### Identification of constitutive binding sites via T-KDE

#### *Overview*

**Figure 2:**
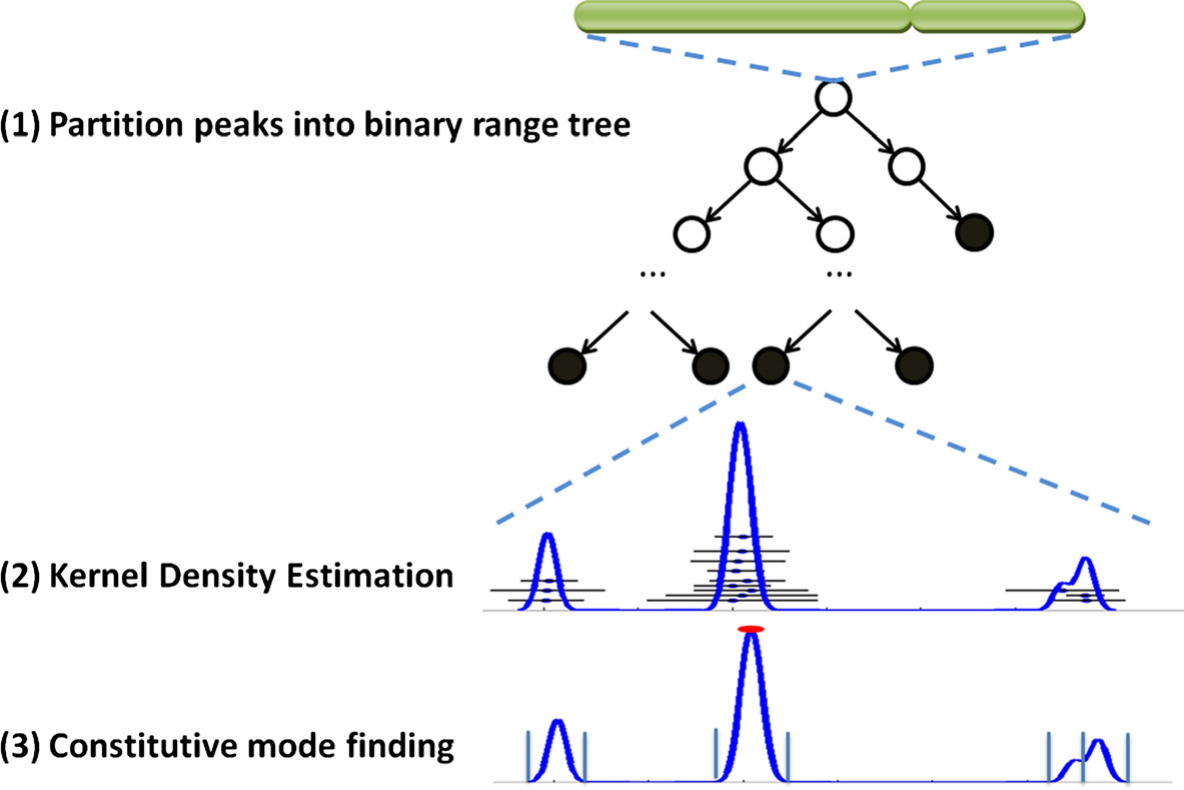
**A schematic overview of T-KDE.As input, T-KDE uses the locations of peak centers (defined by chromosome and coordinate), not the sequence reads.** Step 1: order the peak centers for a TF from all cell lines together and partition them into subsets (terminal nodes) using a binary range tree algorithm. Solid circles indicate terminal nodes. Step 2: apply KDE to estimate a density function for each terminal node. Horizontal lines represent ChIP-seq peaks with dots indicating their centers. The blue curve is the estimated density function. Step 3: apply a mode finding algorithm to each terminal node’s density estimate to identify the modal regions associated with each local maximum. The density function shown has four local maxima (the rightmost two almost coincide); a horizontal red bar marks the constitutive modal region and seven vertical lines mark boundaries of the modal regions.

For ChIP-seq datasets from multiple cell lines, T-KDE identifies genomic regions where peak centers occur. T-KDE starts by partitioning the locations of peak centers into subsets, called terminal nodes, using a binary range tree algorithm (step 1 in Figure 2). For each terminal node, T-KDE uses kernel density estimation to estimate a probability density using all peak centers in the node, the relative frequency with which a peak center will occur near each location along the portion of the genome spanned by the terminal node (step 2). In step 3, for each node, T-KDE finds all local maxima and minima of the estimated probability density function and uses them to define modal regions. The location of each local maximum is taken as a “binding site” location. In this analysis, we required that a modal region contained peak centers from at least two data sets from the same cell line. This requirement is meant to reduce false positives (but could be relaxed). A binding site is declared constitutive when its modal region contains peak centers from at least two replicate datasets per cell line for more than 90% (an arbitrary cutoff that the user can specify) of the available cell lines. Below, we describe each step in more details; the algorithm is provided in Additional file 2.

#### *Binary range tree*

A binary range tree is an algorithm that produces a structure with all data points stored in the leaves (terminal nodes) of the tree for efficient data retrieval and manipulation [25]. In our application, we construct a separate range tree for each chromosome. Initially, all peak centers on the chromosome (from all ChIP-seq data sets for the given TF) are ordered from the smallest to the largest according their genomic locations and placed in the top node. Then, the midrange (mean of the minimum and maximum locations) is used to partition the peak centers into two sub-nodes: the left sub-node contains peak centers whose locations are less than the mid range whereas the right sub-node contains peak centers whose locations equal or exceed the midrange. This process continues recursively within each sub-node until a stopping criterion is satisfied. In our case, a sub-node becomes a terminal node when further partitioning it would result in one or two of its children nodes containing peak centers for fewer than 90% of available cell lines. Although each terminal node in our tree contains peak centers from at least 90% of the cell lines, each terminal node may contain zero, one, or more constitutive binding sites as determined by the subsequent KDE analysis and mode finding.

#### *Kernel density estimation*

Kernel density estimation provides a way to estimate the probability density function of a random variable without assuming a particular parametric form [15,26,27]. For *N* independent samples {*x*_1_, *x*_2_, …, *x*_*N*_} drawn from the same unknown distribution with density *f*, a kernel density estimate of *f* at any point *x*, $\hat{f}\left(x\right)$, is given by:

(1) $$\hat{f}\left(x\right)=\frac{1}{N}\sum_{i=1}^{N}\frac{1}{N}k\left(\frac{x-x_{i}}{h}\right),$$

where *h* represents the bandwidth, a user-defined tuning parameter that controls the smoothness of the resulting estimate. The kernel *K*(•) is a symmetric (not necessarily positive) function that integrates to one, i.e., ∫ *K*(*x*)*dx* = 1. The kernel function serves to smear the probability mass of each data point across a local region.

T-KDE uses the Gaussian kernel, the density function for a Gaussian random variable with mean zero and variance one, defined as:

(2) $$K\left(x\right)={\left(2\pi \right)}^{-1/2}\exp\left\{-\left(x^{2}/2\right)\right\}$$

With this kernel, each term in the sum of equation (1) is a Gaussian density with mean *x*_*i*_ and standard deviation *h*. Thus, equation (1) states that the estimate $\;\hat{f}\left(x\right)$ at any location *x* is formed by averaging contributions from Gaussian densities with standard deviation *h* and means at the observed peak centers. The basic operations of kernel density estimation used by T-KDE have been modified directly from the KDE Toolbox for Matlab [28].

#### *Mode finding in Gaussian mixture models*

To find local maxima and minima of the estimated density function, we adapted a fixed-point iterative search scheme [29]. Our kernel density estimate is an equally weighted mixture of Gaussian densities where the mean of each component is an observed peak center. Such a Gaussian mixture has, at most, as many local maxima as it has components. If peak centers are far apart relative to the bandwidth, each peak center will yield a local maximum. If peak centers are close relative to bandwidth, a local maximum must be between their smallest and largest smallest locations. Thus, within each terminal node, we can use a “hill-climbing” algorithm starting from every peak center to locate all the local maxima and minima. Once we find a location whose gradient is zero using Newton’s method, we use a second derivative test to determine whether it is a maximum or a minimum. Modal regions are defined as extending from the observed peak center farthest to the left of the local maximum but no farther than the next local minimum to the similarly delimited observed peak center farthest to the right. (With this definition, modal regions containing a single peak center have width zero.)

#### *Gene ontology*

We used DAVID [30] to analyze gene ontology (GO). We assigned a constitutive binding site to a gene(s) if the site was located within ±5kb from the gene’s transcription start site using the UCSC refGenes model (hg19). All unique genes that were within the distance were included in the GO analysis.

## Results

### Utility of the binary range tree

Without initial data partition using the binary range tree, KDE analysis and mode finding on even a single chromosome is computationally prohibitive; estimating the density, rather than finding the local maxima/minima, is the bottleneck. For the CTCF datasets, analysis of chromosome 1 took less than half an hour with the binary range tree compared to more than 5 days without it (in Additional file 1: Table S4). The locations of sites declared constitutive using KDE with and without the binary range tree were nearly identical (in Additional file 3: Figure S1).

### Bandwidth and bin width selection

The bandwidth (*h*) of a kernel in KDE estimation and the bin width in the binning approach are tuning parameters whose choice influences each method’s performance in locating binding sites (see Method section). Thus, selection of an appropriate bandwidth or bin width is crucial to accurate identification of binding sites. We systematically tested several different choices for their performance in identifying binding sites using the 132 CTCF ChIP-seq datasets (55 unique cell lines with two or more replicate experiments). We used both binning and T-KDE to identify binding site locations chromosome by chromosome, declaring a binding site constitutive if the corresponding bin or modal region contained peak centers from at least two replicate datasets per cell line for more than 90% of available cell lines. Both the total number of binding sites and the number of constitutive binding sites identified depended on the method (binning vs. T-KDE) and on the value of the corresponding tuning parameter (Table 1). For T-KDE, as expected, the total number of modal regions decreased as the bandwidth increased since a larger bandwidth results fewer but broader modal regions; however, the number of constitutive binding sites remains relatively unchanged. For binning, the number of total binding sites also decreased with increasing bin width because the number of bins, hence, the number of possible binding sites, decreased with increasing bin width, however, the number of constitutive binding sites increased with bin width because wider bins accumulate adjacent binding sites into the same bin, wrongly declaring several non-constitutive sites amalgamated into the same bin as constitutive.

**Table 1:** Observed number of CTCF binding sites on 23 chromosomes

**Bandwidth or bin width (bp)**	**T-KDE**		**Binning**	
	**Number of declared sites**		**Number of declared sites**	
	**Total**	**Constitutive**	**Total**	**Constitutive**
100	142,087	21,812	200,907	2,815
200	133,194	21,884	178,992	11,114
300	128,303	21,834	169,610	14,543
400	124,552	21,750	163,191	16,593
500	121,303	21,690	158,106	17,687
600	118,408	21,606	154,369	18,267
700	115,859	21,523	151,016	18,911
800	113,530	21,464	148,453	19,370
900	111,222	21,375	146,293	19,511
1,000	109,188	21,314	144,005	19,803

Applying the motif-based approach to the same 132 CTCF ChIP-seq data sets with the same criteria (a binding site must be present in at least two replicate datasets per cell line and a constitutive binding site being present in more than 90% of the cell lines) identified 17,575 constitutive CTCF binding sites (the canonical 16-bp motif site). We regarded those motif-based constitutive CTCF biding sites as an “alloyed gold standard”. We have high confidence in a CTCF binding site identified by the motif-based approach because binding at the exact same motif location is detected in more than 90% of cell lines. On the other hand, the motif-based approach is imperfect as it may fail to identify low affinity or indirect binding sites. The motif-based approach could also overlook constitutive sites if the length of peak sequence scanned (200 bp around peak centers in our application) is too short to cover the actual binding site.

**Figure 3:**
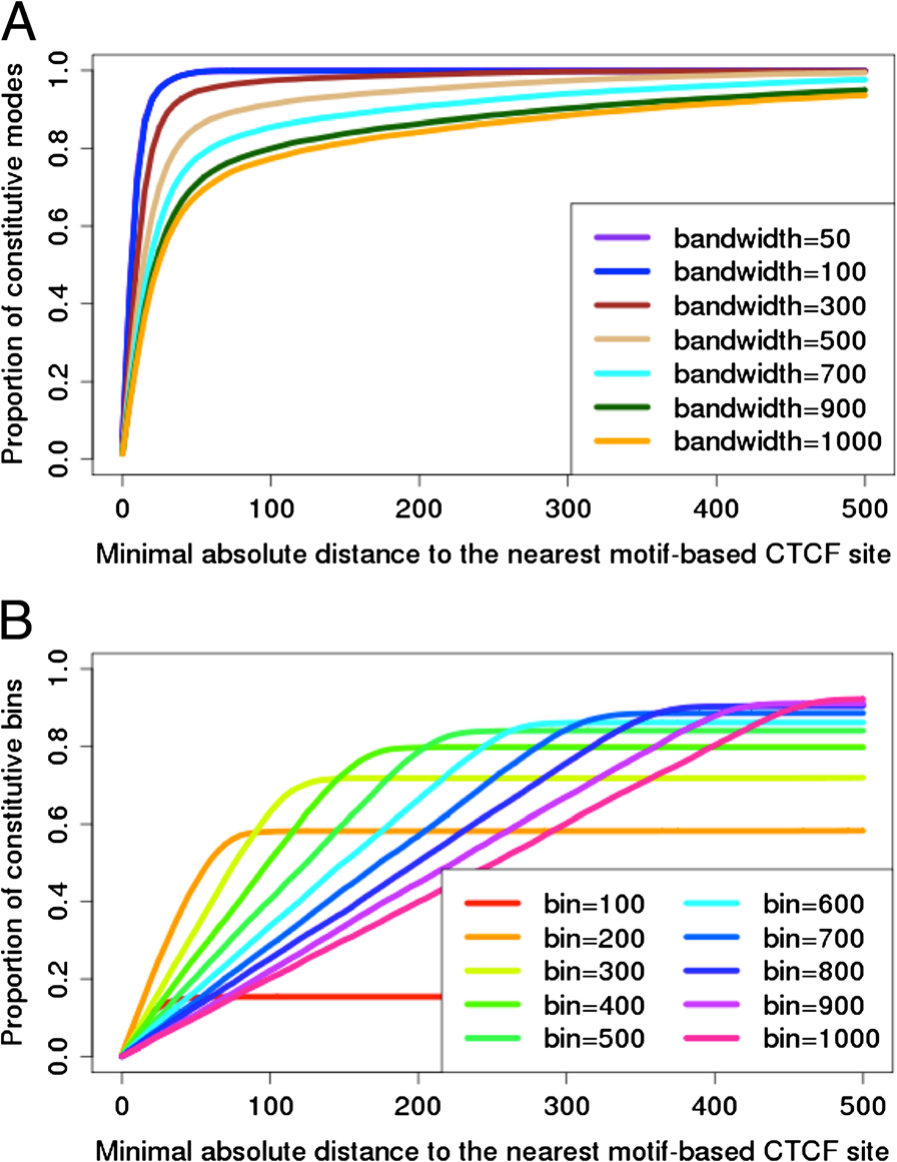
**Performance of T-KDE and binning. (A)** Proportion of T-KDE-declared constitutive CTCF binding sites whose distance from the nearest motif-based constitutive CTCF binding site on 23 chromosomes is less than distance *d* plotted as a function of *d* for various bandwidths. **(B)** Proportion of binning-declared constitutive CTCF binding sites whose distance from nearest motif-based constitutive CTCF binding site on 23 chromosomes is less than distance *d* plotted as a function of *d* for various bin widths.

To compare the locations of constitutive CTCF binding sites from T-KDE and from binning to the locations of our motif-based constitutive CTCF binding sites. We plotted the proportion of constitutive binding sites identified by each method that are less than distance *d* from the nearest motif-based constitutive CTCF binding site as a function of distance *d* (T-KDE in Figure 3(A): binning in Figure 3(B)).

For T-KDE with bandwidths smaller than 500 bp, all CTCF binding sites declared constitutive are within 200 bp of their nearest motif-based constitutive CTCF binding sites. For a bandwidth of 100 bp, more than 90% of the T-KDE-declared constitutive CTCF binding sites are within 20 bp of the nearest motif-based constitutive CTCF binding sites and nearly all are within 70 bp. For bandwidths exceeding 500 bp, performance deteriorates though roughly 90% of the T-KDE-declared constitutive binding sites are still within 500 bp from their nearest motif-based counterpart.

The results from Table 1 and Figure 3 strongly suggest that changing the bandwidth with T-KDE has little impact on the number of constitutive binding sites identified but a greater impact on their locations. On the other hand, changing the bin width with the binning approach has an impact on both the number of constitutive binding sites identified and on their locations. Our results also suggest that, for CTCF, a bandwidth near 100 bp and a bin width near 400 bp may be the optimal values for T-KDE and for the binning method, respectively. Although derived from CTCF comparisons, we believe these choices of bandwidth or bin width should be applicable to other factors whose ChIP-seq peak length distributions are similar to those of CTCF.

Comparing Figure 3(A) and 3(B) also reveals that the accuracy of T-KDE for locating constitutive binding sites is generally far superior to that of the binning approach. In particular, the optimal bandwidth of 100 bp was more accurate in locating constitutive binding sites than the optimal bin width of 400 bp. Consequently, for our remaining analyses, we focus on T-KDE using a bandwidth of 100 bp.

### T-KDE versus Binning

**Figure 4:**
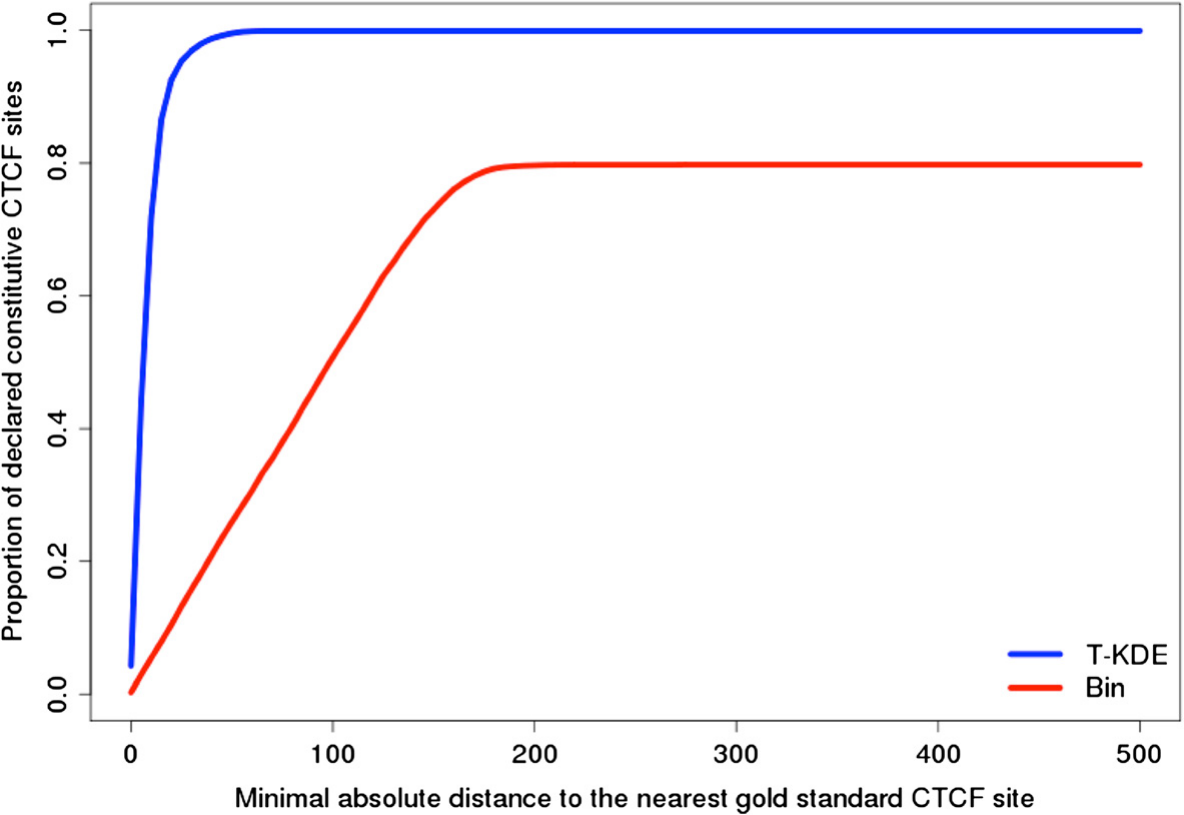
**Proportion of T-KDE declared versus bin declared constitutive CTCF binding sites in the entire genome whose distance from nearest motif-based constitutive CTCF binding site are less than distance *d *plotted as a function of *d*.** Separate curves for T-KDE with bandwidth of 100 bp and bin with size of 400 bp.

Using our motif-based constitutive CTCF binding sites as the reference, we believe that the optimal bandwidth for T-KDE is 100 bp and the optimal bin size for binning method is 400 bp. We plotted the proportion of constitutive binding sites identified by each method that are less than distance from the nearest motif-based constitutive CTCF binding site as a function of distance *d* (Figure 4). As shown in Figure 4, T-KDE is much more accurate in locating constitutive CTCF binding sites than binning method.

### Constitutive sites found by T-KDE but not by the motif-based approach

Only 25 of the 17,575 motif-based constitutive CTCF binding sites were farther than 70 bp from the nearest constitutive CTCF binding sites identified by T-KDE. Furthermore, T-KDE declared an additional 4,237 CTCF binding sites as constitutive that the motif-based approach missed. Among those 4,237 sites, the motif-based approach failed to detect 312 because no sub-sequence in the corresponding peaks reached the motif significance cutoff. (The motif-based approach did not declare any of these as a binding site in any of the cell lines). The remaining additional constitutive sites found by T-KDE were found by the motif-based approach in a majority of cell lines but not in enough cell lines to reach the required 90%. When the true binding sites are not located near the center of some peaks and/or the peak sequences used in motif scan are not long enough to cover the actual motif, a motif-based approach would miss the site. T-DKE, however, is unaffected by these issues. Because it uses peak centers from all cell lines to identify the center of mass of each modal region as the binding site, some misalignment or displacement among ChIP-seq peaks is tolerated. Thus, T-KDE is capable of identifying constitutive binding sites that are bound by a protein either directly or indirectly.

**Figure 5:**
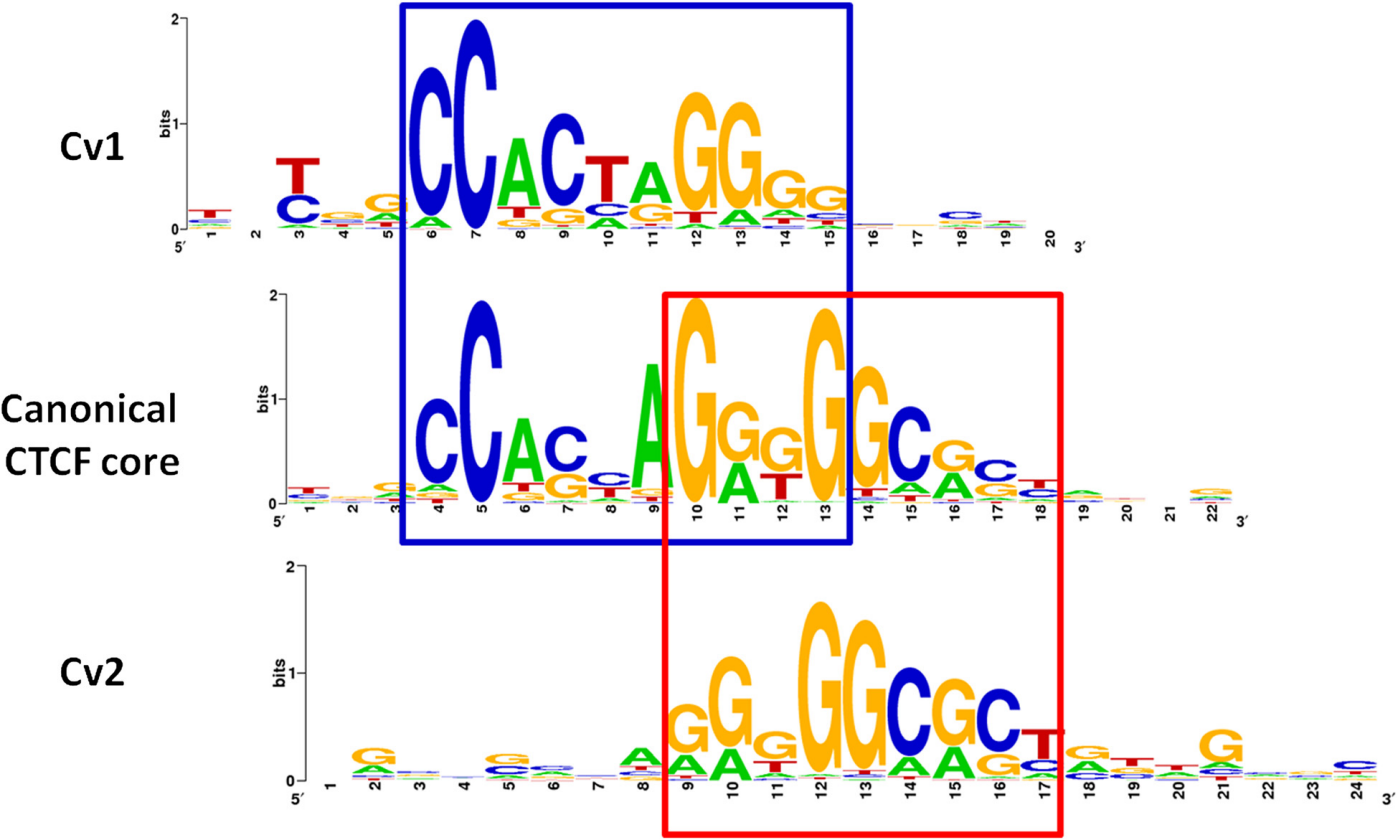
**Motif logos of CTCF motif variants in comparison to the canonical core CTCF motif.** The regions that are aligned to the core CTCF motif are highlighted in red and blue boxes.

Motif discovery using GADEM [24] on the 312 constitutive sites identified by T-KDE where no canonical CTCF motif was found yielded two new motif variants (Figure 5) of the canonical core CTCF motif. We named these motifs as core variant motifs 1 and 2 (Cv1 and Cv2), respectively. Cv2 was the dominant motif found in ~65% of the 312 sequences whereas Cv1 was found in ~35% of the sequences. Compared to the canonical core CTCF motif, these motif variants lacked information at either the 5′- or the 3′-end. This feature may explain their lack of motif significance when compared to the canonical core CTCF motif. Both variants are likely authentic, as they are highly centrally distributed along the 200 bp peak sequences (not shown). It is also likely that such variant motifs are not limited to those 312 constitutive CTCF sites. The two new motifs along with those discovered recently [31,32] add to the complexity of the CTCF code.

### Analysis of constitutive binding sites for 22 factors

We applied T-KDE using a bandwidth of 100 bp to 22 factors with ChIP-seq data available from ENCODE for multiple cell lines with replicates. The number of declared binding sites (modal regions) among these TFs ranged from 30,000 to over 900,000, and the number constitutive binding sites ranged from a few to over 20,000 (Table 2). Constitutive binding sites identified for TFs that were studied in fewer than 10 cell lines, especially when the number of constitutive binding sites identified is relatively small, have a high likelihood of being false positives. As data from additional cell lines becomes available, some sites now declared constitutive could fail to meet the necessary criterion. Thus, we focus attention on the possible biological roles of constitutive binding sites for TFs with more than 1,000 declared constitutive binding sites based on 9 or more cell lines.

**Table 2:** Binding sites throughout the entire genome identified by T-KDE for 22 transcription factors

**Protein**	**Available cell lines**	**Constitutive sites**	**Total sites**	**Proportion**
CTCF	55	21,812	142,087	15.35%
RAD21	9	15,337	101,434	15.12%
GABP	9	1,392	19,444	7.16%
CREB1	6	1,069	16,744	6.38%
YY1	11	2,524	52,252	4.83%
NRSF	15	1,794	40,066	4.48%
TAF1	13	1,208	27,842	4.34%
ELF1	7	860	25,991	3.31%
ZBTB33	6	465	14,429	3.22%
USF1	9	1,172	46,829	2.50%
SRF	7	231	10,263	2.25%
Pol II	19	4,733	261,043	1.81%
MAX	10	1,223	84,862	1.44%
EGR1	6	331	29,793	1.11%
SIN3A	9	116	29,446	0.39%
CEBPB	7	70	62,332	0.11%
TEAD4	8	32	58,134	0.06%
SP1	6	29	46,502	0.06%
ATF3	6	4	9,394	0.04%
P300	10	32	118,602	0.03%
TCF12	7	2	66,517	0.00%
JUND	8	2	51,580	0.00%

Besides CTCF and RAD21, Pol II exhibited a large number of constitutive binding sites (~4,700), although they represented only a small proportion of the total Pol II binding sites. Gene ontology (GO) analysis of the genes containing constitutive Pol II binding sites in their proximal promoters suggests that those Pol II target genes are highly enriched with biological processes such as metabolism, biosynthesis and cell cycle (Table 3). Similarly, genes with constitutive binding sites for other TFs are highly enriched in certain biological processes. These include GABP, NRSF, TAF1, etc. (in Additional file 1: Tables S5-S19). Together, those results suggest that binding sites declared constitutive by T-KDE are connected to important biological processes.

**Table 3:** Top ten GO processes for constitutive Pol II target genes

**Biological process**	**Multiple testing adjusted *p*-value**
Cellular metabolic process	8.2 × 10^-177^
Primary metabolic process	1.2 × 10^-121^
Macromolecule metabolic process	2.8 × 10^-114^
Nitrogen compound metabolic process	8.8 × 10^-87^
Cell cycle	1.0 × 10^-46^
Biosynthetic process	6.3 × 10^-46^
Establishment of protein localization	5.2 × 10^-40^
Organelle organization	1.4 × 10^-37^
Cell cycle process	9.1 × 10^-35^
Ribonucleo protein complex biogenesis	2.5 × 10^-33^

## Discussion

Binding sites that are occupied by a protein regardless of the cell or tissue type seem likely to have a distinct role compared to binding sites for the same protein that are occupied more selectively – the constitutive nature of the binding should signify something of fundamental import. Our earlier work using motif-based analysis found that constitutive CTCF binding sites, especially those near RAD21 sites, are highly enriched in CTCF-mediated chromatin interactions [33] and those interactions are predominately within topological domains, not between them [4]. Consequently, we hypothesized that the constitutive CTCF binding sites may be involved in maintaining and/or establishing chromatin structures that are common among most human cell types [3]. Those earlier findings indicate to us that constitutive binding sites for other TFs may have unique biological roles.

The ENCODE consortium has generated more than 1,000 ChIP-seq protein-binding datasets for more than 100 proteins in multiple cell lines, and the data continue to expand. Discovering the locations and functions the genomic loci that are constitutively bound by each of the proteins is potentially important. However, computational methods for locating constitutive binding sites when the protein does not bind directly to DNA are still lacking. One challenge is that the ChIP-seq peak data are low-resolution, and the technology is unable pinpoint exact genomic binding locations.

To fill this gap, we developed an efficient and effective approach, T-KDE which takes as input locations of peak centers from multiple ChIP-seq data sets and returns estimates of the locations of binding sites and declares them constitutive or not. T-KDE combines a binary range tree algorithm, a kernel density estimator, and a mode finding algorithm. Using data on CTCF binding, we found that T-KDE was superior at locating constitutive binding sites compared to a naïve approach based on binning and that T-KDE performed well compared to the motif-based approach. For example, all motif-based constitutive CTCF binding sites were included in the constitutive CTCF binding sites identified by T-KDE. Furthermore, T-KDE identified additional 4,237 constitutive CTCF binding sites that the motif-based approach failed to detect. This result highlights a major advantage of T-KDE compared to both the motif-based and binning approaches: regardless of whether binding is direct or indirect and whether an adequate motif model is known, T-KDE accurately estimates the locations of constitutive binding sites by identifying genomic regions where the centers of ChIP-seq peaks from multiple datasets lie in close proximity. Accurate binding locations are necessary for subsequent functional analysis and discovery. We applied T-KDE to locate constitutive binding sites, if present, for 22 TFs that had replicate ChIP-seq data sets for at least 6 cell lines available from ENCODE, and we used gene ontology analysis to establish possible biological functions for some of those TFs.

KDE-based methods different from ours have been applied to ChIP-seq reads for peak calling [17] and nucleosome positioning [18]. Additionally, KDE-based method has been applied to motif locations for detection of regions locally enriched with transcription factor binding sites [19]. Our goal is different: we use the locations of ChIP-seq peak centers from multiple cell lines (from as few as 6 to as many as 132, in this case) to infer the location of constitutive binding sites. In addition, our method has unique features. Our method first recursively partitions the locations of peak centers into subgroups (terminal nodes) using a binary range tree algorithm. The partitioning stops whenever either of the two would-be child nodes contains peak centers from fewer than 90% (a user-specified choice) of available cell lines. The KDE analysis and subsequent mode finding is carried out on each terminal node, one at a time. The partitioning guarantees that more than 90% of cell lines are represented in every terminal node; however, a terminal node may still contain zero, one or more constitutive binding sites depending on the spread of the peak centers present — making KDE and subsequent mode-finding necessary for localizing modal regions. Binding site locations are declared at local maxima within modal regions. Our use of the binary range tree before applying KDE and mode-finding makes our algorithm novel and efficient.

One reviewer suggested an alternative procedure (in Additional file 2: Algorithm S3) using the peak-finding algorithm MACS [34]. The procedure involves applying MACS in its default parameters to a combined BAM file from the original ChIP-seq reads data (also in BAM format) from the multiple cell lines. The peaks with low variation in log (read count + 1) within ±50 bp from the MACS summit are considered constitutive. We compared this procedure with T-KDE and a binding-based method and showed that T-KDE was far superior to this alternative procedure (details in Additional file 4: Supplementary text).

Although T-KDE can be applied to ChIP-seq data from any number of cell lines, caution must be excised when interpreting a result from only a few cell lines. Because the property of being constitutive requires binding to the same locus in a variety of cell types, the number and diversity (or lineage) of cell lines/types providing data to the algorithm would be expected to have a strong influence on the biological trustworthiness of any result.

For *N* peak centers, KDE followed by mode-finding has a computational complexity of *O*(*Nlog*_2_*N*) [28,29]*.* When *N* is large as in our CTCF dataset (*N* = ~ 690, 000 for chromosome 1), the process becomes computationally prohibitive. After initial data partitioning by a binary range tree into a set of terminal nodes indexed by *i*, each with *N* peak centers, complexity is greatly reduced to   ∑ _*i*_*O*(*N*_*i*_*log*_2_*N*_*i*_). Consequently, T-KDE reduces the computational time for CTCF on chromosome 1 from days to within an hour. We envision that parallelization of our T-KDE algorithm at the node level would further reduce the computational time. A potential cost is that partitioning all peak centers onto terminal nodes before the KDE analysis and mode finding might destroy a constitutive binding site by splitting it between two adjacent nodes. This problem appears to arise rarely or not at all as we observed that the performance of T-KDE was nearly identical to that of KDE omitting the initial partitioning. We attribute this similarity, in part, to our stopping criterion for partitioning.

Generally, the choice of the bandwidth for KDE can exhibit a strong influence on the shape of the estimated density: small bandwidths yielding spiky estimates and large bandwidths yielding overly flattened ones. Yet, in our comparisons when locating constitutive CTCF binding sites, bandwidths from 100 to 400 bp uncovered similar numbers of constitutive CTCF binding sites and the distribution of the distances from T-KDE-declared sites to the nearest motif-declared sites did not change much with bandwidth. We believe that a bandwidth of 100 to 400 bp may be optimal for most TF binding sites with narrow peaks (100-1,000 bp). Automatic selection of the optimal bandwidth would be desirable, but optimal bandwidth selection based statistical criteria such as the mean integrated squared error [26] did not work well with the CTCF data. That process, which involved maximizing a “pseudo-likelihood” combined with a leave-one-out cross-validation approach [28] was computationally expensive and selected a large bandwidth of 1,293 bp that did not locate constitutive binding sites as well as our preferred 100 bp bandwidth did.

Although designed for identifying constitutive binding sites for a protein using ChIP-seq data from multiple cell lines, our method could also be used to identify genomic loci that have concentrations of different protein binding sites (“hot spots”), and conversely “cold spots”, using multiple protein ChIP-seq data for the cell line.

## Conclusions

In conclusion, we developed efficient and accurate method, T-KDE, to locate constitutive protein binding sites using ChIP-seq peak centers from multiple cell lines. T-KDE combines a binary range tree algorithm, a non-parametric kernel density estimator, and a mode finding algorithm. We showed that, for CTCF data, our method is relatively robust to the choice of bandwidth and is highly accurate when compared to the identification of constitutive binding sites through motif analysis. Application of T-KDE to 22 proteins with ChIP-seq data from multiple cell lines located substantial numbers of constitutive binding sites for some TFs but almost none for others. For TFs with large numbers of constitutive binding sites, GO analysis suggests that these sites are biological meaningful. As additional TF binding sites ChIP-seq datasets become available in more cell lines and for more TFs, our method will prove to be essential for identifying their constitutive binding sites.

## Availability and requirements

Project Name: T-KDE

Project homepage: http://www.niehs.nih.gov/research/resources/software/biostatistics/t-kde/index.cfm

Operating system: Unix

Programming language: Matlab

Other requirements: N/A

License: This work is made available under the GPL v3.

Any restrictions to use by non-academics: none

## Abbreviations

ChIP-Chip: Chromatin immunoprecipitation followed by microarray; ChIP-seq: Chromatin immunoprecipitation followed by sequencing; CTCF: CCCTC binding factor; GABP: GA-binding protein; GO: Gene ontology; KDE: Kernel density estimation; MACS: Model-based analysis of ChIP-seq; NRSF: Neuron-restrictive silencer transcription factor, also known as REST; Pol II: Polymerase II; RAD21: Homolog (*S. pombe*); TAF1: TATA box binding protein-associated factor 1; TF: Transcription factor.

## Competing interests

The authors declare that they have no competing interests.

## Authors’ contributions

LL conceived the study, YL, and LL performed the analyses, and YL, LL, and DMU were all involved in study design, planning analyses, and the interpretation of results. All authors contributed to writing and revising the manuscript, and all approved the final manuscript for publication.

## Supplementary Material

Additional file 1: Supplementary Tables S1-19 Cell lines contributing ChIP-seq data for each of the 22 transcription factors. Click here for file

Additional file 2 **Outlines of algorithms.** T-KDE. Click here for file

Additional file 3: Supplementary Figure S1 Proportion of TKDE-declared versus KDE-declared constitutive CTCF binding sites whose distance from nearest motif-based constitutive CTCF binding site on 23 chromosomesare less than distance d plotted as a function of d for various bandwidths.Separate curves for T-KDE with bandwidth of 100 bps and for the same density estimation algorithm without the binary range tree pre-processing. Click here for file

Additional file 4 **Additional results comparing various methods.** T-KDE: A method for genome-wide identification of constitutive protein binding sites from multiple ChIP-seq data sets. Click here for file
