# Supplementary material for: T-KDE: a method for genome-wide identification of constitutive protein binding sites from multiple ChIP-seq data sets
Source: BMC Genomics. 2014 Jan 15;15:27. doi: 10.1186/1471-2164-15-27 (PMC3903014; doi:10.1186/1471-2164-15-27)
Supplement: Additional file 1: Supplementary Tables S1-19 — Cell lines contributing ChIP-seq data for each of the 22 transcription factors. [file 1471-2164-15-27-S1.docx]

**Supplementary Table 1:** Cell lines contributing ChIP-seq data for each of the 22 transcription factors.

| TF | Available cell lines | No. Cell lines  with >=2 replicates | Total No. replicate data sets used | Cell lines |
| --- | --- | --- | --- | --- |
| CTCF | 63 | 55 | 132 | A549, Ag04449, Ag04450, Ag09309, Ag09319, Ag10803, Aoaf, Be2c, Bj, Caco2, Ecc1, Gm06990, Gm12801, Gm12864, Gm12865, Gm12866, Gm12867, Gm12868, Gm12869, Gm12870, Gm12871, Gm12872, Gm12873, Gm12874, Gm12875, Gm12878, H1hesc, Hac, Hasp, Hbmec, Hcfaa, Hcm, Hcpe, Hct116, Hee, Hek293, Helas3, Hepg2, Hff, Hffmyc, Hl60, Hmec, Hmf, Hpaf, Hpf, Hre, Hrpe, Huvec, Hvmf, Imr90, K562, Mcf7, Nb4, Nhdfneo, Nhek, Nhlf, Rptec, Saec, Sknsh, Sknshra, T47d, Werirb1, Wi38 |
| Pol II | 31 | 19 | 78 | A549, Ecc1, Gm10847, Gm12878, Gm12891, Gm12892, Gm15510, Gm18505, Gm18526, Gm18951, Gm19099, Gm19193, H1hesc, H1neurons, Hct116, Hek293, Helas3, Hepg2, Hl60, Huvec, Imr90, K562, Mcf10aes, Nb4, Panc1, Pbde, Pfsk1, Raji, Sknmc, Sknsh, U87 |
| NRSF | 15 | 15 | 44 | A549, Ecc1, Gm12878, H1hesc, H1neurons, Hct116, Helas3, Hepg2, Hl60, K562, Mcf7, Panc1, Pfsk1, Sknsh, U87 |
| TAD1 | 13 | 13 | 26 | A549, Ecc1, Gm12878, Gm12891, Gm12892, H1hesc, H1neurons, Helas3, Hepg2, K562, Mcf7, Pfsk1, Sknsh |
| YY1 | 12 | 11 | 28 | A549, Ecc1, Gm12878, Gm12891, Gm12892, H1hesc, Hct116, Hepg2, K562, Nt2d1, Sknsh, Sknshra |
| MAX | 12 | 10 | 25 | A549, Ecc1, Gm12878, H1hesc, Hct116, Helas3, Hepg2, Huvec, K562, Mcf7, Nb4, Sknsh |
| P300 | 11 | 10 | 25 | A549, Ecc1, Gm12878, H1hesc, Helas3, Hepg2, K562, Mcf7, Sknsh, Sknshra, T47d |
| RAD21 | 12 | 9 | 23 | A549, Ecc1, Gm12878, H1hesc, Hct116, Helas3, Hepg2, Imr90, K562, Mcf7, Sknsh, Sknshra |
| SIN3A | 10 | 9 | 19 | A549, Gm12878, H1hesc, Hct116, Hepg2, K562, Mcf7, Panc1, Pfsk1, Sknsh |
| USF1 | 9 | 9 | 22 | A549, Ecc1, Gm12878, H1hesc, Hct116, Hepg2, K562, Sknsh, Sknshra |
| GABP | 9 | 9 | 18 | A549, Gm12878, H1hesc, Helas3, Hepg2, Hl60, K562, Mcf7, Sknsh |
| TEAD4 | 8 | 8 | 16 | A549, Ecc1, H1hesc, Hct116, Hepg2, K562, Mcf7, Sknsh |
| JUND | 10 | 8 | 19 | A549, Gm12878, H1hesc, Hct116, Helas3, Hepg2, K562, Mcf7, Sknsh, T47d |
| CEBPB | 10 | 7 | 18 | A549, Ecc1, Gm12878, H1hesc, Hct116, Helas3, Hepg2, Imr90, K562, Mcf7 |
| SRF | 7 | 7 | 16 | Ecc1, Gm12878, H1hesc, Hct116, Hepg2, K562, Mcf7 |
| TCF12 | 7 | 7 | 14 | A549, Ecc1, Gm12878, H1hesc, Hepg2, Mcf7, Sknsh |
| ELF1 | 7 | 7 | 14 | A549, Gm12878, Hct116, Hepg2, K562, Mcf7, Sknsh |
| ZBTB33 | 6 | 6 | 14 | A549, Gm12878, Hct116, Hepg2, K562, Sknsh |
| EGR1 | 6 | 6 | 13 | Ecc1, Gm12878, H1hesc, Hct116, K562, Mcf7 |
| ATF3 | 6 | 6 | 13 | A549, Gm12878, H1hesc, Hct116, Hepg2, K562 |
| SP1 | 6 | 6 | 12 | A549, Gm12878, H1hesc, Hct116, Hepg2, K562 |
| CREB1 | 6 | 6 | 12 | A549, Ecc1, Gm12878, H1hesc, Hepg2, K562 |

**Supplementary Table 2:** List of unique identifiers for the transcription-factor data sets used.

| wgEncodeHaibTfbsA549Ctcfsc5916Pcr1xDex100nmPkRep1.broadPeak |
| --- |
| wgEncodeHaibTfbsA549Ctcfsc5916Pcr1xDex100nmPkRep2.broadPeak |
| wgEncodeHaibTfbsA549Ctcfsc5916Pcr1xEtoh02PkRep1.broadPeak |
| wgEncodeHaibTfbsA549Ctcfsc5916Pcr1xEtoh02PkRep2.broadPeak |
| wgEncodeHaibTfbsEcc1CtcfcV0416102Dm002p1hPkRep1.broadPeak |
| wgEncodeHaibTfbsEcc1CtcfcV0416102Dm002p1hPkRep2.broadPeak |
| wgEncodeHaibTfbsH1hescCtcfsc5916V0416102PkRep1.broadPeak |
| wgEncodeHaibTfbsH1hescCtcfsc5916V0416102PkRep2.broadPeak |
| wgEncodeHaibTfbsHct116CtcfcV0422111PkRep1.broadPeak |
| wgEncodeHaibTfbsHct116CtcfcV0422111PkRep2.broadPeak |
| wgEncodeHaibTfbsHepg2Ctcfsc5916V0416101PkRep1.broadPeak |
| wgEncodeHaibTfbsHepg2Ctcfsc5916V0416101PkRep2.broadPeak |
| wgEncodeHaibTfbsK562CtcfcPcr1xPkRep1.broadPeak |
| wgEncodeHaibTfbsK562CtcfcPcr1xPkRep1V2.broadPeak |
| wgEncodeHaibTfbsK562CtcfcPcr1xPkRep2.broadPeak |
| wgEncodeHaibTfbsMcf7CtcfcV0422111PkRep1.broadPeak |
| wgEncodeHaibTfbsMcf7CtcfcV0422111PkRep2.broadPeak |
| wgEncodeHaibTfbsSknshraCtcfV0416102PkRep1.broadPeak |
| wgEncodeHaibTfbsSknshraCtcfV0416102PkRep2.broadPeak |
| wgEncodeHaibTfbsT47dCtcfsc5916V0416102Dm002p1hPkRep1.broadPeak |
| wgEncodeHaibTfbsT47dCtcfsc5916V0416102Dm002p1hPkRep2.broadPeak |
| wgEncodeSydhTfbsA549CtcfbIggrabPk.narrowPeak |
| wgEncodeSydhTfbsGm12878Ctcfsc15914c20StdPk.narrowPeak |
| wgEncodeSydhTfbsImr90CtcfbIggrabPk.narrowPeak |
| wgEncodeSydhTfbsK562CtcfbIggrabPk.narrowPeak |
| wgEncodeSydhTfbsSknshCtcfbIggrabPk.narrowPeak |
| wgEncodeUwTfbsA549CtcfStdPkRep1.narrowPeak |
| wgEncodeUwTfbsA549CtcfStdPkRep2.narrowPeak |
| wgEncodeUwTfbsAg04449CtcfStdPkRep1.narrowPeak |
| wgEncodeUwTfbsAg04449CtcfStdPkRep2.narrowPeak |
| wgEncodeUwTfbsAg04450CtcfStdPkRep1.narrowPeak |
| wgEncodeUwTfbsAg04450CtcfStdPkRep2.narrowPeak |
| wgEncodeUwTfbsAg09309CtcfStdPkRep1.narrowPeak |
| wgEncodeUwTfbsAg09309CtcfStdPkRep2.narrowPeak |
| wgEncodeUwTfbsAg09319CtcfStdPkRep1.narrowPeak |
| wgEncodeUwTfbsAg09319CtcfStdPkRep2.narrowPeak |
| wgEncodeUwTfbsAg10803CtcfStdPkRep1.narrowPeak |
| wgEncodeUwTfbsAg10803CtcfStdPkRep2.narrowPeak |
| wgEncodeUwTfbsAoafCtcfStdPkRep1.narrowPeak |
| wgEncodeUwTfbsAoafCtcfStdPkRep2.narrowPeak |
| wgEncodeUwTfbsBe2cCtcfStdPkRep1.narrowPeak |
| wgEncodeUwTfbsBe2cCtcfStdPkRep2.narrowPeak |
| wgEncodeUwTfbsBjCtcfStdPkRep1.narrowPeak |
| wgEncodeUwTfbsBjCtcfStdPkRep2.narrowPeak |
| wgEncodeUwTfbsCaco2CtcfStdPkRep1.narrowPeak |
| wgEncodeUwTfbsCaco2CtcfStdPkRep2.narrowPeak |
| wgEncodeUwTfbsGm06990CtcfStdPkRep1.narrowPeak |
| wgEncodeUwTfbsGm06990CtcfStdPkRep2.narrowPeak |
| wgEncodeUwTfbsGm12801CtcfStdPkRep1.narrowPeak |
| wgEncodeUwTfbsGm12864CtcfStdPkRep1.narrowPeak |
| wgEncodeUwTfbsGm12864CtcfStdPkRep2.narrowPeak |
| wgEncodeUwTfbsGm12864CtcfStdPkRep3.narrowPeak |
| wgEncodeUwTfbsGm12865CtcfStdPkRep1.narrowPeak |
| wgEncodeUwTfbsGm12865CtcfStdPkRep2.narrowPeak |
| wgEncodeUwTfbsGm12865CtcfStdPkRep3.narrowPeak |
| wgEncodeUwTfbsGm12866CtcfStdPkRep1.narrowPeak |
| wgEncodeUwTfbsGm12866CtcfStdPkRep2.narrowPeak |
| wgEncodeUwTfbsGm12867CtcfStdPkRep1.narrowPeak |
| wgEncodeUwTfbsGm12867CtcfStdPkRep2.narrowPeak |
| wgEncodeUwTfbsGm12868CtcfStdPkRep1.narrowPeak |
| wgEncodeUwTfbsGm12868CtcfStdPkRep2.narrowPeak |
| wgEncodeUwTfbsGm12869CtcfStdPkRep1.narrowPeak |
| wgEncodeUwTfbsGm12869CtcfStdPkRep2.narrowPeak |
| wgEncodeUwTfbsGm12870CtcfStdPkRep1.narrowPeak |
| wgEncodeUwTfbsGm12870CtcfStdPkRep2.narrowPeak |
| wgEncodeUwTfbsGm12871CtcfStdPkRep1.narrowPeak |
| wgEncodeUwTfbsGm12871CtcfStdPkRep2.narrowPeak |
| wgEncodeUwTfbsGm12872CtcfStdPkRep1.narrowPeak |
| wgEncodeUwTfbsGm12872CtcfStdPkRep2.narrowPeak |
| wgEncodeUwTfbsGm12872CtcfStdPkRep3.narrowPeak |
| wgEncodeUwTfbsGm12873CtcfStdPkRep1.narrowPeak |
| wgEncodeUwTfbsGm12873CtcfStdPkRep2.narrowPeak |
| wgEncodeUwTfbsGm12873CtcfStdPkRep3.narrowPeak |
| wgEncodeUwTfbsGm12874CtcfStdPkRep1.narrowPeak |
| wgEncodeUwTfbsGm12874CtcfStdPkRep2.narrowPeak |
| wgEncodeUwTfbsGm12875CtcfStdPkRep1.narrowPeak |
| wgEncodeUwTfbsGm12875CtcfStdPkRep2.narrowPeak |
| wgEncodeUwTfbsGm12878CtcfStdPkRep1.narrowPeak |
| wgEncodeUwTfbsGm12878CtcfStdPkRep2.narrowPeak |
| wgEncodeUwTfbsHacCtcfStdPkRep1.narrowPeak |
| wgEncodeUwTfbsHacCtcfStdPkRep2.narrowPeak |
| wgEncodeUwTfbsHaspCtcfStdPkRep1.narrowPeak |
| wgEncodeUwTfbsHaspCtcfStdPkRep2.narrowPeak |
| wgEncodeUwTfbsHbmecCtcfStdPkRep1.narrowPeak |
| wgEncodeUwTfbsHbmecCtcfStdPkRep2.narrowPeak |
| wgEncodeUwTfbsHcfaaCtcfStdPkRep1.narrowPeak |
| wgEncodeUwTfbsHcmCtcfStdPkRep1.narrowPeak |
| wgEncodeUwTfbsHcmCtcfStdPkRep2.narrowPeak |
| wgEncodeUwTfbsHcpeCtcfStdPkRep1.narrowPeak |
| wgEncodeUwTfbsHcpeCtcfStdPkRep2.narrowPeak |
| wgEncodeUwTfbsHct116CtcfStdPkRep1.narrowPeak |
| wgEncodeUwTfbsHct116CtcfStdPkRep2.narrowPeak |
| wgEncodeUwTfbsHeeCtcfStdPkRep1.narrowPeak |
| wgEncodeUwTfbsHeeCtcfStdPkRep2.narrowPeak |
| wgEncodeUwTfbsHek293CtcfStdPkRep1.narrowPeak |
| wgEncodeUwTfbsHek293CtcfStdPkRep2.narrowPeak |
| wgEncodeUwTfbsHelas3CtcfStdPkRep1.narrowPeak |
| wgEncodeUwTfbsHelas3CtcfStdPkRep2.narrowPeak |
| wgEncodeUwTfbsHepg2CtcfStdPkRep1.narrowPeak |
| wgEncodeUwTfbsHepg2CtcfStdPkRep2.narrowPeak |
| wgEncodeUwTfbsHffCtcfStdPkRep1.narrowPeak |
| wgEncodeUwTfbsHffmycCtcfStdPkRep1.narrowPeak |
| wgEncodeUwTfbsHffmycCtcfStdPkRep2.narrowPeak |
| wgEncodeUwTfbsHl60CtcfStdPkRep1.narrowPeak |
| wgEncodeUwTfbsHmecCtcfStdPkRep1.narrowPeak |
| wgEncodeUwTfbsHmecCtcfStdPkRep2.narrowPeak |
| wgEncodeUwTfbsHmfCtcfStdPkRep1.narrowPeak |
| wgEncodeUwTfbsHmfCtcfStdPkRep2.narrowPeak |
| wgEncodeUwTfbsHpafCtcfStdPkRep1.narrowPeak |
| wgEncodeUwTfbsHpafCtcfStdPkRep2.narrowPeak |
| wgEncodeUwTfbsHpfCtcfStdPkRep1.narrowPeak |
| wgEncodeUwTfbsHpfCtcfStdPkRep2.narrowPeak |
| wgEncodeUwTfbsHreCtcfStdPkRep1.narrowPeak |
| wgEncodeUwTfbsHreCtcfStdPkRep2.narrowPeak |
| wgEncodeUwTfbsHrpeCtcfStdPkRep1.narrowPeak |
| wgEncodeUwTfbsHrpeCtcfStdPkRep2.narrowPeak |
| wgEncodeUwTfbsHuvecCtcfStdPkRep1.narrowPeak |
| wgEncodeUwTfbsHuvecCtcfStdPkRep2.narrowPeak |
| wgEncodeUwTfbsHvmfCtcfStdPkRep1.narrowPeak |
| wgEncodeUwTfbsHvmfCtcfStdPkRep2.narrowPeak |
| wgEncodeUwTfbsK562CtcfStdPkRep1.narrowPeak |
| wgEncodeUwTfbsK562CtcfStdPkRep2.narrowPeak |
| wgEncodeUwTfbsMcf7CtcfStdPkRep1.narrowPeak |
| wgEncodeUwTfbsMcf7CtcfStdPkRep2.narrowPeak |
| wgEncodeUwTfbsNb4CtcfStdPkRep1.narrowPeak |
| wgEncodeUwTfbsNhdfneoCtcfStdPkRep1.narrowPeak |
| wgEncodeUwTfbsNhdfneoCtcfStdPkRep2.narrowPeak |
| wgEncodeUwTfbsNhekCtcfStdPkRep1.narrowPeak |
| wgEncodeUwTfbsNhekCtcfStdPkRep2.narrowPeak |
| wgEncodeUwTfbsNhlfCtcfStdPkRep1.narrowPeak |
| wgEncodeUwTfbsRptecCtcfStdPkRep1.narrowPeak |
| wgEncodeUwTfbsRptecCtcfStdPkRep2.narrowPeak |
| wgEncodeUwTfbsSaecCtcfStdPkRep1.narrowPeak |
| wgEncodeUwTfbsSaecCtcfStdPkRep2.narrowPeak |
| wgEncodeUwTfbsSknshraCtcfStdPkRep1.narrowPeak |
| wgEncodeUwTfbsSknshraCtcfStdPkRep2.narrowPeak |
| wgEncodeUwTfbsWerirb1CtcfStdPkRep1.narrowPeak |
| wgEncodeUwTfbsWerirb1CtcfStdPkRep2.narrowPeak |
| wgEncodeUwTfbsWi38CtcfStdPkRep1.narrowPeak |
| wgEncodeUwTfbsWi38CtcfStdPkRep2.narrowPeak |
|  |
| wgEncodeHaibTfbsA549Pol2Pcr2xDex100nmPkRep1.broadPeak |
| wgEncodeHaibTfbsA549Pol2Pcr2xDex100nmPkRep2.broadPeak |
| wgEncodeHaibTfbsA549Pol2Pcr2xEtoh02PkRep1.broadPeak |
| wgEncodeHaibTfbsA549Pol2Pcr2xEtoh02PkRep2.broadPeak |
| wgEncodeHaibTfbsEcc1Pol2V0416102Dm002p1hPkRep1.broadPeak |
| wgEncodeHaibTfbsEcc1Pol2V0416102Dm002p1hPkRep2.broadPeak |
| wgEncodeHaibTfbsGm12878Pol24h8Pcr1xPkRep1.broadPeak |
| wgEncodeHaibTfbsGm12878Pol24h8Pcr1xPkRep2.broadPeak |
| wgEncodeHaibTfbsGm12878Pol2Pcr2xPkRep1.broadPeak |
| wgEncodeHaibTfbsGm12878Pol2Pcr2xPkRep2.broadPeak |
| wgEncodeHaibTfbsGm12891Pol24h8Pcr1xPkRep1.broadPeak |
| wgEncodeHaibTfbsGm12891Pol24h8Pcr1xPkRep2.broadPeak |
| wgEncodeHaibTfbsGm12891Pol2Pcr1xPkRep1.broadPeak |
| wgEncodeHaibTfbsGm12891Pol2Pcr1xPkRep2.broadPeak |
| wgEncodeHaibTfbsGm12892Pol24h8V0416102PkRep1.broadPeak |
| wgEncodeHaibTfbsGm12892Pol24h8V0416102PkRep2.broadPeak |
| wgEncodeHaibTfbsGm12892Pol2V0416102PkRep1.broadPeak |
| wgEncodeHaibTfbsGm12892Pol2V0416102PkRep2.broadPeak |
| wgEncodeHaibTfbsH1hescPol24h8V0416102PkRep1.broadPeak |
| wgEncodeHaibTfbsH1hescPol24h8V0416102PkRep2.broadPeak |
| wgEncodeHaibTfbsH1hescPol2V0416102PkRep1.broadPeak |
| wgEncodeHaibTfbsH1hescPol2V0416102PkRep2.broadPeak |
| wgEncodeHaibTfbsH1neuronsPol24h8V0422111PkRep1.broadPeak |
| wgEncodeHaibTfbsH1neuronsPol24h8V0422111PkRep2.broadPeak |
| wgEncodeHaibTfbsHct116Pol24h8V0416101PkRep1.broadPeak |
| wgEncodeHaibTfbsHct116Pol24h8V0416101PkRep2.broadPeak |
| wgEncodeHaibTfbsHelas3Pol2Pcr1xPkRep1.broadPeak |
| wgEncodeHaibTfbsHelas3Pol2Pcr1xPkRep2.broadPeak |
| wgEncodeHaibTfbsHepg2Pol24h8V0416102PkRep1.broadPeak |
| wgEncodeHaibTfbsHepg2Pol24h8V0416102PkRep2.broadPeak |
| wgEncodeHaibTfbsHepg2Pol2Pcr2xPkRep1.broadPeak |
| wgEncodeHaibTfbsHepg2Pol2Pcr2xPkRep2.broadPeak |
| wgEncodeHaibTfbsHl60Pol24h8V0422111PkRep1.broadPeak |
| wgEncodeHaibTfbsHl60Pol24h8V0422111PkRep2.broadPeak |
| wgEncodeHaibTfbsHuvecPol24h8V0416101PkRep1.broadPeak |
| wgEncodeHaibTfbsHuvecPol24h8V0416101PkRep2.broadPeak |
| wgEncodeHaibTfbsHuvecPol2Pcr1xPkRep1.broadPeak |
| wgEncodeHaibTfbsHuvecPol2Pcr1xPkRep2.broadPeak |
| wgEncodeHaibTfbsK562Pol24h8V0416101PkRep1.broadPeak |
| wgEncodeHaibTfbsK562Pol24h8V0416101PkRep2.broadPeak |
| wgEncodeHaibTfbsK562Pol2V0416101PkRep1.broadPeak |
| wgEncodeHaibTfbsK562Pol2V0416101PkRep2.broadPeak |
| wgEncodeHaibTfbsPanc1Pol24h8V0416101PkRep1.broadPeak |
| wgEncodeHaibTfbsPanc1Pol24h8V0416101PkRep2.broadPeak |
| wgEncodeHaibTfbsPfsk1Pol24h8V0416101PkRep1.broadPeak |
| wgEncodeHaibTfbsPfsk1Pol24h8V0416101PkRep2.broadPeak |
| wgEncodeHaibTfbsSknmcPol24h8V0416101PkRep1.broadPeak |
| wgEncodeHaibTfbsSknmcPol24h8V0416101PkRep2.broadPeak |
| wgEncodeHaibTfbsSknshPol24h8V0416101PkRep1.broadPeak |
| wgEncodeHaibTfbsSknshPol24h8V0416101PkRep2.broadPeak |
| wgEncodeHaibTfbsU87Pol24h8V0416101PkRep1.broadPeak |
| wgEncodeHaibTfbsU87Pol24h8V0416101PkRep1V2.broadPeak |
| wgEncodeHaibTfbsU87Pol24h8V0416101PkRep2.broadPeak |
| wgEncodeHaibTfbsU87Pol24h8V0416101PkRep2V2.broadPeak |
| wgEncodeSydhTfbsA549Pol2s2IggrabPk.narrowPeak |
| wgEncodeSydhTfbsGm10847Pol2IggmusPk.narrowPeak |
| wgEncodeSydhTfbsGm12878Pol2IggmusPk.narrowPeak |
| wgEncodeSydhTfbsGm12878Pol2s2IggmusPk.narrowPeak |
| wgEncodeSydhTfbsGm12878Pol2StdPk.narrowPeak |
| wgEncodeSydhTfbsGm12891Pol2IggmusPk.narrowPeak |
| wgEncodeSydhTfbsGm12892Pol2IggmusPk.narrowPeak |
| wgEncodeSydhTfbsGm15510Pol2IggmusPk.narrowPeak |
| wgEncodeSydhTfbsGm18505Pol2IggmusPk.narrowPeak |
| wgEncodeSydhTfbsGm18526Pol2IggmusPk.narrowPeak |
| wgEncodeSydhTfbsGm18951Pol2IggmusPk.narrowPeak |
| wgEncodeSydhTfbsGm19099Pol2IggmusPk.narrowPeak |
| wgEncodeSydhTfbsGm19193Pol2IggmusPk.narrowPeak |
| wgEncodeSydhTfbsHct116Pol2UcdPk.narrowPeak |
| wgEncodeSydhTfbsHek293Pol2StdPk.narrowPeak |
| wgEncodeSydhTfbsHelas3Pol2s2IggrabPk.narrowPeak |
| wgEncodeSydhTfbsHelas3Pol2StdPk.narrowPeak |
| wgEncodeSydhTfbsHepg2Pol2ForsklnStdPk.narrowPeak |
| wgEncodeSydhTfbsHepg2Pol2IggrabPk.narrowPeak |
| wgEncodeSydhTfbsHepg2Pol2PravastStdPk.narrowPeak |
| wgEncodeSydhTfbsHepg2Pol2s2IggrabPk.narrowPeak |
| wgEncodeSydhTfbsHuvecPol2StdPk.narrowPeak |
| wgEncodeSydhTfbsImr90Pol2IggrabPk.narrowPeak |
| wgEncodeSydhTfbsK562Pol2Ifna30StdPk.narrowPeak |
| wgEncodeSydhTfbsK562Pol2Ifna6hStdPk.narrowPeak |
| wgEncodeSydhTfbsK562Pol2Ifng30StdPk.narrowPeak |
| wgEncodeSydhTfbsK562Pol2Ifng6hStdPk.narrowPeak |
| wgEncodeSydhTfbsK562Pol2IggmusPk.narrowPeak |
| wgEncodeSydhTfbsK562Pol2s2IggrabPk.narrowPeak |
| wgEncodeSydhTfbsK562Pol2s2StdPk.narrowPeak |
| wgEncodeSydhTfbsK562Pol2StdPk.narrowPeak |
| wgEncodeSydhTfbsMcf10aesPol2Etoh01StdPk.narrowPeak |
| wgEncodeSydhTfbsMcf10aesPol2TamStdPk.narrowPeak |
| wgEncodeSydhTfbsNb4Pol2StdPk.narrowPeak |
| wgEncodeSydhTfbsPbdePol2UcdPk.narrowPeak |
| wgEncodeSydhTfbsRajiPol2UcdPk.narrowPeak |
|  |
| wgEncodeHaibTfbsA549NrsfV0422111Etoh02PkRep1.broadPeak |
| wgEncodeHaibTfbsA549NrsfV0422111Etoh02PkRep2.broadPeak |
| wgEncodeHaibTfbsEcc1NrsfV0422111PkRep1.broadPeak |
| wgEncodeHaibTfbsEcc1NrsfV0422111PkRep2.broadPeak |
| wgEncodeHaibTfbsGm12878NrsfPcr1xPkRep1.broadPeak |
| wgEncodeHaibTfbsGm12878NrsfPcr1xPkRep2.broadPeak |
| wgEncodeHaibTfbsGm12878NrsfPcr2xPkRep1.broadPeak |
| wgEncodeHaibTfbsGm12878NrsfPcr2xPkRep2.broadPeak |
| wgEncodeHaibTfbsH1hescNrsfV0416102PkRep1.broadPeak |
| wgEncodeHaibTfbsH1hescNrsfV0416102PkRep2.broadPeak |
| wgEncodeHaibTfbsH1neuronsNrsfV0422111PkRep1.broadPeak |
| wgEncodeHaibTfbsH1neuronsNrsfV0422111PkRep2.broadPeak |
| wgEncodeHaibTfbsHct116NrsfV0422111PkRep1.broadPeak |
| wgEncodeHaibTfbsHct116NrsfV0422111PkRep2.broadPeak |
| wgEncodeHaibTfbsHelas3NrsfPcr1xPkRep1.broadPeak |
| wgEncodeHaibTfbsHelas3NrsfPcr1xPkRep2.broadPeak |
| wgEncodeHaibTfbsHepg2NrsfPcr2xPkRep1.broadPeak |
| wgEncodeHaibTfbsHepg2NrsfPcr2xPkRep2.broadPeak |
| wgEncodeHaibTfbsHepg2NrsfV0416101PkRep1.broadPeak |
| wgEncodeHaibTfbsHepg2NrsfV0416101PkRep2.broadPeak |
| wgEncodeHaibTfbsHl60NrsfV0422111PkRep1.broadPeak |
| wgEncodeHaibTfbsHl60NrsfV0422111PkRep2.broadPeak |
| wgEncodeHaibTfbsK562NrsfV0416102PkRep1.broadPeak |
| wgEncodeHaibTfbsK562NrsfV0416102PkRep2.broadPeak |
| wgEncodeHaibTfbsMcf7NrsfV0422111PkRep1.broadPeak |
| wgEncodeHaibTfbsMcf7NrsfV0422111PkRep2.broadPeak |
| wgEncodeHaibTfbsPanc1NrsfPcr2xPkRep1.broadPeak |
| wgEncodeHaibTfbsPanc1NrsfPcr2xPkRep2.broadPeak |
| wgEncodeHaibTfbsPanc1NrsfV0416101PkRep1.broadPeak |
| wgEncodeHaibTfbsPanc1NrsfV0416101PkRep2.broadPeak |
| wgEncodeHaibTfbsPanc1NrsfV0422111PkRep1.broadPeak |
| wgEncodeHaibTfbsPanc1NrsfV0422111PkRep2.broadPeak |
| wgEncodeHaibTfbsPfsk1NrsfPcr2xPkRep1.broadPeak |
| wgEncodeHaibTfbsPfsk1NrsfPcr2xPkRep2.broadPeak |
| wgEncodeHaibTfbsPfsk1NrsfV0416101PkRep1.broadPeak |
| wgEncodeHaibTfbsPfsk1NrsfV0416101PkRep2.broadPeak |
| wgEncodeHaibTfbsSknshNrsfPcr2xPkRep1.broadPeak |
| wgEncodeHaibTfbsSknshNrsfPcr2xPkRep2.broadPeak |
| wgEncodeHaibTfbsSknshNrsfV0416101PkRep1.broadPeak |
| wgEncodeHaibTfbsSknshNrsfV0416101PkRep2.broadPeak |
| wgEncodeHaibTfbsU87NrsfPcr2xPkRep1.broadPeak |
| wgEncodeHaibTfbsU87NrsfPcr2xPkRep1V2.broadPeak |
| wgEncodeHaibTfbsU87NrsfPcr2xPkRep2.broadPeak |
| wgEncodeHaibTfbsU87NrsfPcr2xPkRep2V2.broadPeak |
|  |
| wgEncodeHaibTfbsA549Taf1V0422111Etoh02PkRep1.broadPeak |
| wgEncodeHaibTfbsA549Taf1V0422111Etoh02PkRep2.broadPeak |
| wgEncodeHaibTfbsEcc1Taf1V0422111PkRep1.broadPeak |
| wgEncodeHaibTfbsEcc1Taf1V0422111PkRep2.broadPeak |
| wgEncodeHaibTfbsGm12878Taf1Pcr1xPkRep1.broadPeak |
| wgEncodeHaibTfbsGm12878Taf1Pcr1xPkRep2.broadPeak |
| wgEncodeHaibTfbsGm12891Taf1Pcr1xPkRep1.broadPeak |
| wgEncodeHaibTfbsGm12891Taf1Pcr1xPkRep2.broadPeak |
| wgEncodeHaibTfbsGm12892Taf1V0416102PkRep1.broadPeak |
| wgEncodeHaibTfbsGm12892Taf1V0416102PkRep2.broadPeak |
| wgEncodeHaibTfbsH1hescTaf1V0416102PkRep1.broadPeak |
| wgEncodeHaibTfbsH1hescTaf1V0416102PkRep2.broadPeak |
| wgEncodeHaibTfbsH1neuronsTaf1V0422111PkRep1.broadPeak |
| wgEncodeHaibTfbsH1neuronsTaf1V0422111PkRep2.broadPeak |
| wgEncodeHaibTfbsHelas3Taf1Pcr1xPkRep1.broadPeak |
| wgEncodeHaibTfbsHelas3Taf1Pcr1xPkRep2.broadPeak |
| wgEncodeHaibTfbsHepg2Taf1Pcr2xPkRep1.broadPeak |
| wgEncodeHaibTfbsHepg2Taf1Pcr2xPkRep2.broadPeak |
| wgEncodeHaibTfbsK562Taf1V0416101PkRep1.broadPeak |
| wgEncodeHaibTfbsK562Taf1V0416101PkRep2.broadPeak |
| wgEncodeHaibTfbsMcf7Taf1V0422111PkRep1.broadPeak |
| wgEncodeHaibTfbsMcf7Taf1V0422111PkRep2.broadPeak |
| wgEncodeHaibTfbsPfsk1Taf1V0416101PkRep1.broadPeak |
| wgEncodeHaibTfbsPfsk1Taf1V0416101PkRep2.broadPeak |
| wgEncodeHaibTfbsSknshTaf1V0416101PkRep1.broadPeak |
| wgEncodeHaibTfbsSknshTaf1V0416101PkRep2.broadPeak |
|  |
| wgEncodeHaibTfbsA549Rad21V0422111PkRep1.broadPeak |
| wgEncodeHaibTfbsA549Rad21V0422111PkRep2.broadPeak |
| wgEncodeHaibTfbsEcc1Rad21V0422111PkRep1.broadPeak |
| wgEncodeHaibTfbsEcc1Rad21V0422111PkRep2.broadPeak |
| wgEncodeHaibTfbsGm12878Rad21V0416101PkRep1.broadPeak |
| wgEncodeHaibTfbsGm12878Rad21V0416101PkRep2.broadPeak |
| wgEncodeHaibTfbsH1hescRad21V0416102PkRep1.broadPeak |
| wgEncodeHaibTfbsH1hescRad21V0416102PkRep2.broadPeak |
| wgEncodeHaibTfbsHct116Rad21V0422111PkRep1.broadPeak |
| wgEncodeHaibTfbsHct116Rad21V0422111PkRep2.broadPeak |
| wgEncodeHaibTfbsHepg2Rad21V0416101PkRep1.broadPeak |
| wgEncodeHaibTfbsHepg2Rad21V0416101PkRep2.broadPeak |
| wgEncodeHaibTfbsK562Rad21V0416102PkRep1.broadPeak |
| wgEncodeHaibTfbsK562Rad21V0416102PkRep2.broadPeak |
| wgEncodeHaibTfbsMcf7Rad21V0422111PkRep1.broadPeak |
| wgEncodeHaibTfbsMcf7Rad21V0422111PkRep2.broadPeak |
| wgEncodeHaibTfbsSknshraRad21V0416102PkRep1.broadPeak |
| wgEncodeHaibTfbsSknshraRad21V0416102PkRep2.broadPeak |
| wgEncodeSydhTfbsA549Rad21IggrabPk.narrowPeak |
| wgEncodeSydhTfbsGm12878Rad21IggrabPk.narrowPeak |
| wgEncodeSydhTfbsH1hescRad21IggrabPk.narrowPeak |
| wgEncodeSydhTfbsHelas3Rad21IggrabPk.narrowPeak |
| wgEncodeSydhTfbsHepg2Rad21IggrabPk.narrowPeak |
| wgEncodeSydhTfbsImr90Rad21IggrabPk.narrowPeak |
| wgEncodeSydhTfbsK562Rad21StdPk.narrowPeak |
| wgEncodeSydhTfbsSknshRad21IggrabPk.narrowPeak |
|  |
| wgEncodeHaibTfbsA549GabpV0422111Etoh02PkRep1.broadPeak |
| wgEncodeHaibTfbsA549GabpV0422111Etoh02PkRep2.broadPeak |
| wgEncodeHaibTfbsGm12878GabpPcr2xPkRep1.broadPeak |
| wgEncodeHaibTfbsGm12878GabpPcr2xPkRep2.broadPeak |
| wgEncodeHaibTfbsH1hescGabpPcr1xPkRep1.broadPeak |
| wgEncodeHaibTfbsH1hescGabpPcr1xPkRep2.broadPeak |
| wgEncodeHaibTfbsHelas3GabpPcr1xPkRep1.broadPeak |
| wgEncodeHaibTfbsHelas3GabpPcr1xPkRep2.broadPeak |
| wgEncodeHaibTfbsHepg2GabpPcr2xPkRep1.broadPeak |
| wgEncodeHaibTfbsHepg2GabpPcr2xPkRep2.broadPeak |
| wgEncodeHaibTfbsHl60GabpV0422111PkRep1.broadPeak |
| wgEncodeHaibTfbsHl60GabpV0422111PkRep2.broadPeak |
| wgEncodeHaibTfbsK562GabpV0416101PkRep1.broadPeak |
| wgEncodeHaibTfbsK562GabpV0416101PkRep2.broadPeak |
| wgEncodeHaibTfbsMcf7GabpV0422111PkRep1.broadPeak |
| wgEncodeHaibTfbsMcf7GabpV0422111PkRep2.broadPeak |
| wgEncodeHaibTfbsSknshGabpV0422111PkRep1.broadPeak |
| wgEncodeHaibTfbsSknshGabpV0422111PkRep2.broadPeak |
|  |
| wgEncodeHaibTfbsA549Creb1sc240V0416102Dex100nmPkRep1.broadPeak |
| wgEncodeHaibTfbsA549Creb1sc240V0416102Dex100nmPkRep2.broadPeak |
| wgEncodeHaibTfbsEcc1Creb1sc240V0422111PkRep1.broadPeak |
| wgEncodeHaibTfbsEcc1Creb1sc240V0422111PkRep2.broadPeak |
| wgEncodeHaibTfbsGm12878Creb1sc240V0422111PkRep1.broadPeak |
| wgEncodeHaibTfbsGm12878Creb1sc240V0422111PkRep2.broadPeak |
| wgEncodeHaibTfbsH1hescCreb1sc240V0422111PkRep1.broadPeak |
| wgEncodeHaibTfbsH1hescCreb1sc240V0422111PkRep2.broadPeak |
| wgEncodeHaibTfbsHepg2Creb1sc240V0422111PkRep1.broadPeak |
| wgEncodeHaibTfbsHepg2Creb1sc240V0422111PkRep2.broadPeak |
| wgEncodeHaibTfbsK562Creb1sc240V0422111PkRep1.broadPeak |
| wgEncodeHaibTfbsK562Creb1sc240V0422111PkRep2.broadPeak |
|  |
| wgEncodeHaibTfbsA549Yy1cV0422111Etoh02PkRep1.broadPeak |
| wgEncodeHaibTfbsA549Yy1cV0422111Etoh02PkRep2.broadPeak |
| wgEncodeHaibTfbsEcc1Yy1sc281V0422111PkRep1.broadPeak |
| wgEncodeHaibTfbsEcc1Yy1sc281V0422111PkRep2.broadPeak |
| wgEncodeHaibTfbsGm12878Yy1sc281Pcr1xPkRep1.broadPeak |
| wgEncodeHaibTfbsGm12878Yy1sc281Pcr1xPkRep2.broadPeak |
| wgEncodeHaibTfbsGm12891Yy1sc281V0416101PkRep1.broadPeak |
| wgEncodeHaibTfbsGm12891Yy1sc281V0416101PkRep2.broadPeak |
| wgEncodeHaibTfbsGm12892Yy1V0416101PkRep1.broadPeak |
| wgEncodeHaibTfbsGm12892Yy1V0416101PkRep2.broadPeak |
| wgEncodeHaibTfbsH1hescYy1sc281V0416102PkRep1.broadPeak |
| wgEncodeHaibTfbsH1hescYy1sc281V0416102PkRep2.broadPeak |
| wgEncodeHaibTfbsHct116Yy1sc281V0416101PkRep1.broadPeak |
| wgEncodeHaibTfbsHct116Yy1sc281V0416101PkRep2.broadPeak |
| wgEncodeHaibTfbsHepg2Yy1sc281V0416101PkRep1.broadPeak |
| wgEncodeHaibTfbsHepg2Yy1sc281V0416101PkRep2.broadPeak |
| wgEncodeHaibTfbsK562Yy1sc281V0416101PkRep1.broadPeak |
| wgEncodeHaibTfbsK562Yy1sc281V0416101PkRep2.broadPeak |
| wgEncodeHaibTfbsK562Yy1V0416101PkRep1.broadPeak |
| wgEncodeHaibTfbsK562Yy1V0416101PkRep2.broadPeak |
| wgEncodeHaibTfbsK562Yy1V0416102PkRep1.broadPeak |
| wgEncodeHaibTfbsK562Yy1V0416102PkRep2.broadPeak |
| wgEncodeHaibTfbsSknshraYy1sc281V0416102PkRep1.broadPeak |
| wgEncodeHaibTfbsSknshraYy1sc281V0416102PkRep2.broadPeak |
| wgEncodeHaibTfbsSknshYy1sc281V0422111PkRep1.broadPeak |
| wgEncodeHaibTfbsSknshYy1sc281V0422111PkRep2.broadPeak |
| wgEncodeSydhTfbsGm12878Yy1StdPk.narrowPeak |
| wgEncodeSydhTfbsK562Yy1UcdPk.narrowPeak |
| wgEncodeSydhTfbsNt2d1Yy1UcdPk.narrowPeak |
|  |
| wgEncodeHaibTfbsA549Usf1Pcr1xDex100nmPkRep1.broadPeak |
| wgEncodeHaibTfbsA549Usf1Pcr1xDex100nmPkRep2.broadPeak |
| wgEncodeHaibTfbsA549Usf1Pcr1xEtoh02PkRep1.broadPeak |
| wgEncodeHaibTfbsA549Usf1Pcr1xEtoh02PkRep2.broadPeak |
| wgEncodeHaibTfbsA549Usf1V0422111Etoh02PkRep1.broadPeak |
| wgEncodeHaibTfbsA549Usf1V0422111Etoh02PkRep2.broadPeak |
| wgEncodeHaibTfbsEcc1Usf1V0422111PkRep1.broadPeak |
| wgEncodeHaibTfbsEcc1Usf1V0422111PkRep2.broadPeak |
| wgEncodeHaibTfbsGm12878Usf1Pcr2xPkRep1.broadPeak |
| wgEncodeHaibTfbsGm12878Usf1Pcr2xPkRep2.broadPeak |
| wgEncodeHaibTfbsH1hescUsf1Pcr1xPkRep1.broadPeak |
| wgEncodeHaibTfbsH1hescUsf1Pcr1xPkRep2.broadPeak |
| wgEncodeHaibTfbsHct116Usf1V0422111PkRep1.broadPeak |
| wgEncodeHaibTfbsHct116Usf1V0422111PkRep2.broadPeak |
| wgEncodeHaibTfbsHepg2Usf1Pcr1xPkRep1.broadPeak |
| wgEncodeHaibTfbsHepg2Usf1Pcr1xPkRep2.broadPeak |
| wgEncodeHaibTfbsK562Usf1V0416101PkRep1.broadPeak |
| wgEncodeHaibTfbsK562Usf1V0416101PkRep2.broadPeak |
| wgEncodeHaibTfbsSknshraUsf1sc8983V0416102PkRep1.broadPeak |
| wgEncodeHaibTfbsSknshraUsf1sc8983V0416102PkRep2.broadPeak |
| wgEncodeHaibTfbsSknshUsf1V0422111PkRep1.broadPeak |
| wgEncodeHaibTfbsSknshUsf1V0422111PkRep2.broadPeak |
|  |
| wgEncodeHaibTfbsA549Elf1V0422111Etoh02PkRep1.broadPeak |
| wgEncodeHaibTfbsA549Elf1V0422111Etoh02PkRep2.broadPeak |
| wgEncodeHaibTfbsGm12878Elf1sc631V0416101PkRep1.broadPeak |
| wgEncodeHaibTfbsGm12878Elf1sc631V0416101PkRep2.broadPeak |
| wgEncodeHaibTfbsHct116Elf1V0422111PkRep1.broadPeak |
| wgEncodeHaibTfbsHct116Elf1V0422111PkRep2.broadPeak |
| wgEncodeHaibTfbsHepg2Elf1sc631V0416101PkRep1.broadPeak |
| wgEncodeHaibTfbsHepg2Elf1sc631V0416101PkRep2.broadPeak |
| wgEncodeHaibTfbsK562Elf1sc631V0416102PkRep1.broadPeak |
| wgEncodeHaibTfbsK562Elf1sc631V0416102PkRep2.broadPeak |
| wgEncodeHaibTfbsMcf7Elf1V0422111PkRep1.broadPeak |
| wgEncodeHaibTfbsMcf7Elf1V0422111PkRep2.broadPeak |
| wgEncodeHaibTfbsSknshElf1V0422111PkRep1.broadPeak |
| wgEncodeHaibTfbsSknshElf1V0422111PkRep2.broadPeak |
|  |
| wgEncodeHaibTfbsA549Zbtb33V0422111Etoh02PkRep1.broadPeak |
| wgEncodeHaibTfbsA549Zbtb33V0422111Etoh02PkRep2.broadPeak |
| wgEncodeHaibTfbsGm12878Zbtb33Pcr1xPkRep1.broadPeak |
| wgEncodeHaibTfbsGm12878Zbtb33Pcr1xPkRep2.broadPeak |
| wgEncodeHaibTfbsHct116Zbtb33V0416101PkRep1.broadPeak |
| wgEncodeHaibTfbsHct116Zbtb33V0416101PkRep2.broadPeak |
| wgEncodeHaibTfbsHepg2Zbtb33Pcr1xPkRep1.broadPeak |
| wgEncodeHaibTfbsHepg2Zbtb33Pcr1xPkRep2.broadPeak |
| wgEncodeHaibTfbsHepg2Zbtb33V0416101PkRep1.broadPeak |
| wgEncodeHaibTfbsHepg2Zbtb33V0416101PkRep2.broadPeak |
| wgEncodeHaibTfbsK562Zbtb33Pcr1xPkRep1.broadPeak |
| wgEncodeHaibTfbsK562Zbtb33Pcr1xPkRep2.broadPeak |
| wgEncodeHaibTfbsSknshZbtb33V0422111PkRep1.broadPeak |
| wgEncodeHaibTfbsSknshZbtb33V0422111PkRep2.broadPeak |
|  |
| wgEncodeHaibTfbsEcc1SrfV0422111PkRep1.broadPeak |
| wgEncodeHaibTfbsEcc1SrfV0422111PkRep2.broadPeak |
| wgEncodeHaibTfbsGm12878SrfPcr2xPkRep1.broadPeak |
| wgEncodeHaibTfbsGm12878SrfPcr2xPkRep2.broadPeak |
| wgEncodeHaibTfbsGm12878SrfV0416101PkRep1.broadPeak |
| wgEncodeHaibTfbsGm12878SrfV0416101PkRep2.broadPeak |
| wgEncodeHaibTfbsH1hescSrfPcr1xPkRep1.broadPeak |
| wgEncodeHaibTfbsH1hescSrfPcr1xPkRep2.broadPeak |
| wgEncodeHaibTfbsHct116SrfV0422111PkRep1.broadPeak |
| wgEncodeHaibTfbsHct116SrfV0422111PkRep2.broadPeak |
| wgEncodeHaibTfbsHepg2SrfV0416101PkRep1.broadPeak |
| wgEncodeHaibTfbsHepg2SrfV0416101PkRep2.broadPeak |
| wgEncodeHaibTfbsK562SrfV0416101PkRep1.broadPeak |
| wgEncodeHaibTfbsK562SrfV0416101PkRep2.broadPeak |
| wgEncodeHaibTfbsMcf7SrfV0422111PkRep1.broadPeak |
| wgEncodeHaibTfbsMcf7SrfV0422111PkRep2.broadPeak |
|  |
| wgEncodeHaibTfbsEcc1Egr1V0422111PkRep1.broadPeak |
| wgEncodeHaibTfbsEcc1Egr1V0422111PkRep2.broadPeak |
| wgEncodeHaibTfbsGm12878Egr1Pcr2xPkRep3.broadPeak |
| wgEncodeHaibTfbsGm12878Egr1V0416101PkRep1.broadPeak |
| wgEncodeHaibTfbsGm12878Egr1V0416101PkRep2.broadPeak |
| wgEncodeHaibTfbsH1hescEgr1V0416102PkRep1.broadPeak |
| wgEncodeHaibTfbsH1hescEgr1V0416102PkRep2.broadPeak |
| wgEncodeHaibTfbsHct116Egr1V0422111PkRep1.broadPeak |
| wgEncodeHaibTfbsHct116Egr1V0422111PkRep2.broadPeak |
| wgEncodeHaibTfbsK562Egr1V0416101PkRep1.broadPeak |
| wgEncodeHaibTfbsK562Egr1V0416101PkRep2.broadPeak |
| wgEncodeHaibTfbsMcf7Egr1V0422111PkRep1.broadPeak |
| wgEncodeHaibTfbsMcf7Egr1V0422111PkRep2.broadPeak |
|  |
| wgEncodeHaibTfbsA549MaxV0422111PkRep1.broadPeak |
| wgEncodeHaibTfbsA549MaxV0422111PkRep2.broadPeak |
| wgEncodeHaibTfbsEcc1MaxV0422111PkRep1.broadPeak |
| wgEncodeHaibTfbsEcc1MaxV0422111PkRep2.broadPeak |
| wgEncodeHaibTfbsH1hescMaxV0422111PkRep1.broadPeak |
| wgEncodeHaibTfbsH1hescMaxV0422111PkRep2.broadPeak |
| wgEncodeHaibTfbsHct116MaxV0422111PkRep1.broadPeak |
| wgEncodeHaibTfbsHct116MaxV0422111PkRep2.broadPeak |
| wgEncodeHaibTfbsHepg2MaxV0422111PkRep1.broadPeak |
| wgEncodeHaibTfbsHepg2MaxV0422111PkRep2.broadPeak |
| wgEncodeHaibTfbsK562MaxV0416102PkRep1.broadPeak |
| wgEncodeHaibTfbsK562MaxV0416102PkRep2.broadPeak |
| wgEncodeHaibTfbsMcf7MaxV0422111PkRep1.broadPeak |
| wgEncodeHaibTfbsMcf7MaxV0422111PkRep2.broadPeak |
| wgEncodeHaibTfbsSknshMaxV0422111PkRep1.broadPeak |
| wgEncodeHaibTfbsSknshMaxV0422111PkRep2.broadPeak |
| wgEncodeSydhTfbsA549MaxIggrabPk.narrowPeak |
| wgEncodeSydhTfbsGm12878MaxIggmusPk.narrowPeak |
| wgEncodeSydhTfbsGm12878MaxStdPk.narrowPeak |
| wgEncodeSydhTfbsH1hescMaxUcdPk.narrowPeak |
| wgEncodeSydhTfbsHelas3MaxIggrabPk.narrowPeak |
| wgEncodeSydhTfbsHelas3MaxStdPk.narrowPeak |
| wgEncodeSydhTfbsHepg2MaxIggrabPk.narrowPeak |
| wgEncodeSydhTfbsHuvecMaxStdPk.narrowPeak |
| wgEncodeSydhTfbsK562MaxIggrabPk.narrowPeak |
| wgEncodeSydhTfbsK562MaxStdPk.narrowPeak |
| wgEncodeSydhTfbsNb4MaxStdPk.narrowPeak |
|  |
| wgEncodeHaibTfbsA549Sin3ak20V0422111Etoh02PkRep1.broadPeak |
| wgEncodeHaibTfbsA549Sin3ak20V0422111Etoh02PkRep2.broadPeak |
| wgEncodeHaibTfbsH1hescSin3ak20Pcr1xPkRep1.broadPeak |
| wgEncodeHaibTfbsH1hescSin3ak20Pcr1xPkRep2.broadPeak |
| wgEncodeHaibTfbsHct116Sin3ak20V0422111PkRep1.broadPeak |
| wgEncodeHaibTfbsHct116Sin3ak20V0422111PkRep2.broadPeak |
| wgEncodeHaibTfbsHepg2Sin3ak20Pcr1xPkRep1.broadPeak |
| wgEncodeHaibTfbsHepg2Sin3ak20Pcr1xPkRep2.broadPeak |
| wgEncodeHaibTfbsK562Sin3ak20V0416101PkRep1.broadPeak |
| wgEncodeHaibTfbsK562Sin3ak20V0416101PkRep2.broadPeak |
| wgEncodeHaibTfbsMcf7Sin3ak20V0422111PkRep1.broadPeak |
| wgEncodeHaibTfbsMcf7Sin3ak20V0422111PkRep2.broadPeak |
| wgEncodeHaibTfbsPanc1Sin3ak20V0416101PkRep1.broadPeak |
| wgEncodeHaibTfbsPanc1Sin3ak20V0416101PkRep2.broadPeak |
| wgEncodeHaibTfbsPfsk1Sin3ak20V0416101PkRep1.broadPeak |
| wgEncodeHaibTfbsPfsk1Sin3ak20V0416101PkRep2.broadPeak |
| wgEncodeHaibTfbsSknshSin3ak20V0416101PkRep1.broadPeak |
| wgEncodeHaibTfbsSknshSin3ak20V0416101PkRep2.broadPeak |
| wgEncodeSydhTfbsGm12878Sin3anb6001263IggmusPk.narrowPeak |
| wgEncodeSydhTfbsH1hescSin3anb6001263IggrabPk.narrowPeak |
|  |
| wgEncodeHaibTfbsA549Sp1V0422111Etoh02PkRep1.broadPeak |
| wgEncodeHaibTfbsA549Sp1V0422111Etoh02PkRep2.broadPeak |
| wgEncodeHaibTfbsGm12878Sp1Pcr1xPkRep1.broadPeak |
| wgEncodeHaibTfbsGm12878Sp1Pcr1xPkRep2.broadPeak |
| wgEncodeHaibTfbsH1hescSp1Pcr1xPkRep1.broadPeak |
| wgEncodeHaibTfbsH1hescSp1Pcr1xPkRep2.broadPeak |
| wgEncodeHaibTfbsHct116Sp1V0422111PkRep1.broadPeak |
| wgEncodeHaibTfbsHct116Sp1V0422111PkRep2.broadPeak |
| wgEncodeHaibTfbsHepg2Sp1Pcr1xPkRep1.broadPeak |
| wgEncodeHaibTfbsHepg2Sp1Pcr1xPkRep2.broadPeak |
| wgEncodeHaibTfbsK562Sp1Pcr1xPkRep1.broadPeak |
| wgEncodeHaibTfbsK562Sp1Pcr1xPkRep2.broadPeak |
|  |
| wgEncodeHaibTfbsA549Tead4sc101184V0422111PkRep1.broadPeak |
| wgEncodeHaibTfbsA549Tead4sc101184V0422111PkRep2.broadPeak |
| wgEncodeHaibTfbsEcc1Tead4sc101184V0422111PkRep1.broadPeak |
| wgEncodeHaibTfbsEcc1Tead4sc101184V0422111PkRep2.broadPeak |
| wgEncodeHaibTfbsH1hescTead4sc101184V0422111PkRep1.broadPeak |
| wgEncodeHaibTfbsH1hescTead4sc101184V0422111PkRep2.broadPeak |
| wgEncodeHaibTfbsHct116Tead4sc101184V0422111PkRep1.broadPeak |
| wgEncodeHaibTfbsHct116Tead4sc101184V0422111PkRep2.broadPeak |
| wgEncodeHaibTfbsHepg2Tead4sc101184V0422111PkRep1.broadPeak |
| wgEncodeHaibTfbsHepg2Tead4sc101184V0422111PkRep2.broadPeak |
| wgEncodeHaibTfbsK562Tead4sc101184V0422111PkRep1.broadPeak |
| wgEncodeHaibTfbsK562Tead4sc101184V0422111PkRep2.broadPeak |
| wgEncodeHaibTfbsMcf7Tead4sc101184V0422111PkRep1.broadPeak |
| wgEncodeHaibTfbsMcf7Tead4sc101184V0422111PkRep2.broadPeak |
| wgEncodeHaibTfbsSknshTead4sc101184V0422111PkRep1.broadPeak |
| wgEncodeHaibTfbsSknshTead4sc101184V0422111PkRep2.broadPeak |
|  |
| wgEncodeHaibTfbsA549Cebpbsc150V0422111PkRep1.broadPeak |
| wgEncodeHaibTfbsA549Cebpbsc150V0422111PkRep2.broadPeak |
| wgEncodeHaibTfbsEcc1Cebpbsc150V0422111PkRep1.broadPeak |
| wgEncodeHaibTfbsEcc1Cebpbsc150V0422111PkRep2.broadPeak |
| wgEncodeHaibTfbsGm12878Cebpbsc150V0422111PkRep1.broadPeak |
| wgEncodeHaibTfbsGm12878Cebpbsc150V0422111PkRep2.broadPeak |
| wgEncodeHaibTfbsHct116Cebpbsc150V0422111PkRep1.broadPeak |
| wgEncodeHaibTfbsHct116Cebpbsc150V0422111PkRep2.broadPeak |
| wgEncodeHaibTfbsHepg2Cebpbsc150V0416101PkRep1.broadPeak |
| wgEncodeHaibTfbsHepg2Cebpbsc150V0416101PkRep2.broadPeak |
| wgEncodeHaibTfbsK562Cebpbsc150V0422111PkRep1.broadPeak |
| wgEncodeHaibTfbsK562Cebpbsc150V0422111PkRep2.broadPeak |
| wgEncodeHaibTfbsMcf7Cebpbsc150V0422111PkRep1.broadPeak |
| wgEncodeHaibTfbsMcf7Cebpbsc150V0422111PkRep2.broadPeak |
| wgEncodeSydhTfbsA549CebpbIggrabPk.narrowPeak |
| wgEncodeSydhTfbsH1hescCebpbIggrabPk.narrowPeak |
| wgEncodeSydhTfbsHelas3CebpbIggrabPk.narrowPeak |
| wgEncodeSydhTfbsHepg2CebpbForsklnStdPk.narrowPeak |
| wgEncodeSydhTfbsHepg2CebpbIggrabPk.narrowPeak |
| wgEncodeSydhTfbsImr90CebpbIggrabPk.narrowPeak |
| wgEncodeSydhTfbsK562CebpbIggrabPk.narrowPeak |
|  |
| wgEncodeHaibTfbsA549Atf3V0422111Etoh02PkRep1.broadPeak |
| wgEncodeHaibTfbsA549Atf3V0422111Etoh02PkRep2.broadPeak |
| wgEncodeHaibTfbsGm12878Atf3Pcr1xPkRep1.broadPeak |
| wgEncodeHaibTfbsGm12878Atf3Pcr1xPkRep2.broadPeak |
| wgEncodeHaibTfbsH1hescAtf3V0416102PkRep1.broadPeak |
| wgEncodeHaibTfbsH1hescAtf3V0416102PkRep2.broadPeak |
| wgEncodeHaibTfbsHct116Atf3V0422111PkRep1.broadPeak |
| wgEncodeHaibTfbsHct116Atf3V0422111PkRep2.broadPeak |
| wgEncodeHaibTfbsHepg2Atf3V0416101PkRep1.broadPeak |
| wgEncodeHaibTfbsHepg2Atf3V0416101PkRep2.broadPeak |
| wgEncodeHaibTfbsK562Atf3V0416101PkRep1.broadPeak |
| wgEncodeHaibTfbsK562Atf3V0416101PkRep2.broadPeak |
| wgEncodeSydhTfbsK562Atf3StdPk.narrowPeak |
|  |
| wgEncodeHaibTfbsA549P300V0422111Etoh02PkRep1.broadPeak |
| wgEncodeHaibTfbsA549P300V0422111Etoh02PkRep2.broadPeak |
| wgEncodeHaibTfbsEcc1P300V0422111PkRep1.broadPeak |
| wgEncodeHaibTfbsEcc1P300V0422111PkRep2.broadPeak |
| wgEncodeHaibTfbsGm12878P300Pcr1xPkRep1.broadPeak |
| wgEncodeHaibTfbsGm12878P300Pcr1xPkRep2.broadPeak |
| wgEncodeHaibTfbsH1hescP300V0416102PkRep1.broadPeak |
| wgEncodeHaibTfbsH1hescP300V0416102PkRep2.broadPeak |
| wgEncodeHaibTfbsHepg2P300V0416101PkRep1.broadPeak |
| wgEncodeHaibTfbsHepg2P300V0416101PkRep2.broadPeak |
| wgEncodeHaibTfbsMcf7P300V0422111PkRep1.broadPeak |
| wgEncodeHaibTfbsMcf7P300V0422111PkRep2.broadPeak |
| wgEncodeHaibTfbsSknshP300V0422111PkRep1.broadPeak |
| wgEncodeHaibTfbsSknshP300V0422111PkRep2.broadPeak |
| wgEncodeHaibTfbsSknshraP300V0416102PkRep1.broadPeak |
| wgEncodeHaibTfbsSknshraP300V0416102PkRep2.broadPeak |
| wgEncodeHaibTfbsT47dP300V0416102Dm002p1hPkRep1.broadPeak |
| wgEncodeHaibTfbsT47dP300V0416102Dm002p1hPkRep2.broadPeak |
| wgEncodeSydhTfbsGm12878P300bStdPk.narrowPeak |
| wgEncodeSydhTfbsGm12878P300IggmusPk.narrowPeak |
| wgEncodeSydhTfbsGm12878P300sc584IggmusPk.narrowPeak |
| wgEncodeSydhTfbsHelas3P300sc584sc584IggrabPk.narrowPeak |
| wgEncodeSydhTfbsHepg2P300sc582IggrabPk.narrowPeak |
| wgEncodeSydhTfbsK562P300IggrabPk.narrowPeak |
| wgEncodeSydhTfbsK562P300sc584sc48343IggrabPk.narrowPeak |
| wgEncodeSydhTfbsSknshP300bIggrabPk.narrowPeak |
|  |
| wgEncodeHaibTfbsA549Tcf12V0422111Etoh02PkRep1.broadPeak |
| wgEncodeHaibTfbsA549Tcf12V0422111Etoh02PkRep2.broadPeak |
| wgEncodeHaibTfbsEcc1Tcf12V0422111PkRep1.broadPeak |
| wgEncodeHaibTfbsEcc1Tcf12V0422111PkRep2.broadPeak |
| wgEncodeHaibTfbsGm12878Tcf12Pcr1xPkRep1.broadPeak |
| wgEncodeHaibTfbsGm12878Tcf12Pcr1xPkRep2.broadPeak |
| wgEncodeHaibTfbsH1hescTcf12Pcr1xPkRep1.broadPeak |
| wgEncodeHaibTfbsH1hescTcf12Pcr1xPkRep2.broadPeak |
| wgEncodeHaibTfbsHepg2Tcf12Pcr1xPkRep1.broadPeak |
| wgEncodeHaibTfbsHepg2Tcf12Pcr1xPkRep2.broadPeak |
| wgEncodeHaibTfbsMcf7Tcf12V0422111PkRep1.broadPeak |
| wgEncodeHaibTfbsMcf7Tcf12V0422111PkRep2.broadPeak |
| wgEncodeHaibTfbsSknshTcf12V0422111PkRep1.broadPeak |
| wgEncodeHaibTfbsSknshTcf12V0422111PkRep2.broadPeak |
|  |
| wgEncodeHaibTfbsA549JundV0416102Etoh02PkRep1.broadPeak |
| wgEncodeHaibTfbsA549JundV0416102Etoh02PkRep2.broadPeak |
| wgEncodeHaibTfbsH1hescJundV0416102PkRep1.broadPeak |
| wgEncodeHaibTfbsH1hescJundV0416102PkRep2.broadPeak |
| wgEncodeHaibTfbsHct116JundV0422111PkRep1.broadPeak |
| wgEncodeHaibTfbsHct116JundV0422111PkRep2.broadPeak |
| wgEncodeHaibTfbsHepg2JundPcr1xPkRep1.broadPeak |
| wgEncodeHaibTfbsHepg2JundPcr1xPkRep2.broadPeak |
| wgEncodeHaibTfbsMcf7JundV0422111PkRep1.broadPeak |
| wgEncodeHaibTfbsMcf7JundV0422111PkRep2.broadPeak |
| wgEncodeHaibTfbsSknshJundV0422111PkRep1.broadPeak |
| wgEncodeHaibTfbsSknshJundV0422111PkRep2.broadPeak |
| wgEncodeHaibTfbsT47dJundV0422111PkRep1.broadPeak |
| wgEncodeHaibTfbsT47dJundV0422111PkRep2.broadPeak |
| wgEncodeSydhTfbsGm12878JundIggrabPk.narrowPeak |
| wgEncodeSydhTfbsGm12878JundStdPk.narrowPeak |
| wgEncodeSydhTfbsH1hescJundIggrabPk.narrowPeak |
| wgEncodeSydhTfbsHelas3JundIggrabPk.narrowPeak |
| wgEncodeSydhTfbsHepg2JundIggrabPk.narrowPeak |
| wgEncodeSydhTfbsK562JundIggrabPk.narrowPeak |
| wgEncodeSydhTfbsSknshJundIggrabPk.narrowPeak |

**Supplementary Table 3:** CTCF position weight matrix

| A | 0.00 | 0.00 | 0.00 | 0.00 | 0.00 | 1.00 | 0.17 | 0.00 | 0.49 | 0.17 | 0.21 | 0.00 | 0.00 | 1.00 | 0.00 | 0.24 | 0.61 |
| --- | --- | --- | --- | --- | --- | --- | --- | --- | --- | --- | --- | --- | --- | --- | --- | --- | --- |
| C | 0.46 | 1.00 | 0.00 | 0.00 | 1.00 | 0.00 | 0.16 | 0.00 | 0.00 | 0.46 | 0.74 | 1.00 | 1.00 | 0.00 | 0.10 | 0.25 | 0.20 |
| G | 0.54 | 0.00 | 0.00 | 1.00 | 0.00 | 0.00 | 0.51 | 0.00 | 0.51 | 0.28 | 0.05 | 0.00 | 0.00 | 0.00 | 0.90 | 0.51 | 0.19 |
| T | 0.00 | 0.00 | 1.00 | 0.00 | 0.00 | 0.00 | 0.16 | 1.00 | 0.00 | 0.08 | 0.00 | 0.00 | 0.00 | 0.00 | 0.00 | 0.00 | 0.00 |

Supplementary Table 4: Computational time for 116 CTCF ChIP-seq datasets in 56 cell lines for various bandwidths on chromosome 1 without initial data partitioning using the binary range tree (A) and with initial data partitioning using the binary range tree (B).

(A)

| **Bandwidth** | **Total no. modes** | **Total no. constitutive modes** | **Time in seconds (days)** |
| --- | --- | --- | --- |
| 100 | 42,241 | 2,724 | 456,455.10 (5.3) |
| 200 | 35,051 | 2,760 | 452,962.55 (5.2) |
| 300 | 31,580 | 2,763 | 457,094.32 (5.3) |
| 400 | 29,106 | 2,742 | 449,902.20 (5.2) |

(B)

| **Bandwidth** | **Total no. modes** | **Total no. constitutive modes** | **Time in seconds (minutes)** |
| --- | --- | --- | --- |
| 100 | 42,339 | 2,724 | 1,101.43 (18.36) |
| 200 | 35,181 | 2,756 | 1,067.75 (17.80) |
| 300 | 31,746 | 2,758 | 1,063.29 (17.72) |
| 400 | 29,291 | 2,751 | 1,077.02 (17.95) |

**Supplementary Table 5:** Top 10 significant GO biological processes for constitutive GABP target genes

| **Biological process** | **Multiple testing adjusted *p*-value** |
| --- | --- |
| cellular metabolic process | 2.7×10^-86^ |
| macromolecule metabolic process | 1.3×10^-80^ |
| primary metabolic process | 4.6×10^-65^ |
| nitrogen compound metabolic process | 8.7×10^-47^ |
| ribonucleoprotein complex biogenesis | 1.6×10^-27^ |
| biosynthetic process | 2.1×10^-25^ |
| establishment of protein localization | 6.1×10^-15^ |
| macromolecule localization | 3.0×10^-10^ |
| catabolic process | 1.1×10^-9^ |
| regulation of metabolic process | 1.8×10^-8^ |

**Supplementary Table 6:** Top 10 GO biological processes for constitutive NRSF (Rest) target genes

| **Biological process** | **Multiple testing adjusted *p*-value** |
| --- | --- |
| cell communication | 3.9×10^-9^ |
| secretion by cell | 1.9×10^-6^ |
| transport | 1.3×10^-6^ |
| establishment of localization | 1.7×10^-6^ |
| cell projection organization | 2.5×10^-5^ |
| system process | 3.2×10^-5^ |
| generation of a signal involved in cell-cell signaling | 1.2×10^-4^ |
| adult behavior | 1.2×10^-4^ |
| negative regulation of response to stimulus | 4.6×10^-4^ |
| anatomical structure development | 4.5×10^-4^ |

**Supplementary Table 7:** Top 10 GO biological processes for constitutive TAF1 target genes

| **Biological process** | **Multiple testing adjusted *p*-value** |
| --- | --- |
| cellular metabolic process | 3.6×10^-174^ |
| macromolecule metabolic process | 2.5×10^-124^ |
| primary metabolic process | 4.4×10^-113^ |
| nitrogen compound metabolic process | 1.0×10^-69^ |
| biosynthetic process | 7.9×10^-50^ |
| ribonucleoprotein complex biogenesis | 1.9×10^-38^ |
| establishment of protein localization | 1.9×10^-33^ |
| catabolic process | 1.6×10^-32^ |
| organelle organization | 1.7×10^-26^ |
| macromolecule localization | 7.4×10^-26^ |

**Supplementary Table 8:** Top 10 GO biological processes for constitutive CREB1 target genes

| **Biological process** | **Multiple testing adjusted *p*-value** |
| --- | --- |
| macromolecule metabolic process | 6.6E-31 |
| cellular metabolic process | 7.1E-31 |
| primary metabolic process | 2.1E-22 |
| nitrogen compound metabolic process | 1.8E-15 |
| biosynthetic process | 4.3E-10 |
| ribonucleoprotein complex biogenesis | 4.4E-10 |
| cell cycle process | 3.6E-7 |
| organelle organization | 3.6E-6 |
| cell cycle | 1.8E-5 |
| establishment of protein localization | 4.1E-5 |

**Supplementary Table 9:** Top 10 GO biological processes for constitutive CTCF target genes

| **Biological process** | **Multiple testing adjusted *p*-value** | |
| --- | --- | --- |
| positive regulation of biological process | 2.5E-8 |  |
| positive regulation of cellular process | 1.7E-8 |  |
| anatomical structure development | 1.5E-5 |  |
| multicellular organismal development | 2.0E-5 |  |
| cellular developmental process | 5.7E-5 |  |
| cellular component assembly | 1.3E-4 |  |
| negative regulation of biological process | 1.8E-4 |  |
| positive regulation of metabolic process | 1.8E-4 |  |
| macromolecular complex subunit organization | 1.8E-4 |  |

**Supplementary Table 10:** Top 10 GO biological processes for constitutive EGR1 target genes

| **Biological process** | **Multiple testing adjusted *p*-value** | |
| --- | --- | --- |
| cellular metabolic process | 3.6E-3 |  |
| macromolecule metabolic process | 2.5E-3 |  |
| nitrogen compound metabolic process | 1.4E-2 |  |
| primary metabolic process | 2.9E-2 |  |
| cellular localization | 3.0E-1 |  |
| biosynthetic process | 4.5E-1 |  |
| establishment of localization in cell | 4.2E-1 |  |
| establishment of protein localization | 4.2E-1 |  |
| translational initiation | 5.2E-1 |  |
| catabolic process | 5.2E-1 |  |

**Supplementary Table 11:** Top 10 GO biological processes for constitutive ELF1 target genes

| **Biological process** | **Multiple testing adjusted *p*-value** | |
| --- | --- | --- |
| cellular metabolic process | 1.7E-35 |  |
| macromolecule metabolic process | 2.9E-34 |  |
| primary metabolic process | 1.2E-27 |  |
| nitrogen compound metabolic process | 1.5E-19 |  |
| biosynthetic process | 1.2E-12 |  |
| establishment of protein localization | 2.0E-10 |  |
| ribonucleoprotein complex biogenesis | 2.9E-9 |  |
| establishment of localization in cell | 1.4E-7 |  |
| vesicle-mediated transport | 1.3E-7 |  |
| macromolecule localization | 2.9E-7 |  |

**Supplementary Table 12:** Top 10 GO biological processes for constitutive MAX target genes

| **Biological process** | **Multiple testing adjusted *p*-value** | |
| --- | --- | --- |
| ribonucleoprotein complex biogenesis | 7.2E-40 |  |
| cellular metabolic process | 3.9E-36 |  |
| primary metabolic process | 1.6E-30 |  |
| macromolecule metabolic process | 8.3E-30 |  |
| nitrogen compound metabolic process | 1.5E-28 |  |
| biosynthetic process | 1.4E-16 |  |
| macromolecular complex subunit organization | 6.4E-5 |  |
| establishment of protein localization | 6.1E-5 |  |
| organelle organization | 6.3E-5 |  |
| translational initiation | 9.1E-5 |  |

**Supplementary Table 13:** Top 10 GO biological processes for constitutive Pol II target genes

| **Biological process** | **Multiple testing adjusted *p*-value** | |
| --- | --- | --- |
| cellular metabolic process | 6.5E-141 |  |
| primary metabolic process | 4.4E-96 |  |
| macromolecule metabolic process | 3.0E-96 |  |
| nitrogen compound metabolic process | 5.0E-73 |  |
| cell cycle | 2.2E-39 |  |
| biosynthetic process | 6.1E-33 |  |
| cell cycle process | 1.1E-30 |  |
| organelle organization | 1.3E-30 |  |
| establishment of protein localization | 1.2E-30 |  |
| catabolic process | 1.9E-27 |  |

**Supplementary Table 14:** Top 10 GO biological processes for constitutive RAD21 target genes

| **Biological process** | **Multiple testing adjusted *p*-value** | |
| --- | --- | --- |
| multicellular organismal development | 1.7E-8 |  |
| positive regulation of biological process | 5.7E-8 |  |
| anatomical structure development | 6.7E-8 |  |
| cellular developmental process | 6.8E-7 |  |
| positive regulation of cellular process | 4.8E-6 |  |
| ossification | 7.7E-6 |  |
| anatomical structure morphogenesis | 9.4E-6 |  |
| regulation of multicellular organismal process | 1.5E-4 |  |
| regulation of biological quality | 4.8E-4 |  |
| developmental maturation | 8.7E-4 |  |

**Supplementary Table 15:** Top 10 GO biological processes for constitutive Sin3a target genes

| **Biological process** | **Multiple testing adjusted *p*-value** | |
| --- | --- | --- |
| multicellular organismal development | 1.7E-8 |  |
| positive regulation of biological process | 5.7E-8 |  |
| anatomical structure development | 6.7E-8 |  |
| cellular developmental process | 6.8E-7 |  |
| positive regulation of cellular process | 4.8E-6 |  |
| ossification | 7.7E-6 |  |
| anatomical structure morphogenesis | 9.4E-6 |  |
| regulation of multicellular organismal process | 1.5E-4 |  |
| regulation of biological quality | 4.8E-4 |  |
| developmental maturation | 8.7E-4 |  |

**Supplementary Table 16:** Top 10 GO biological processes for constitutive SRF target genes

| **Biological process** | **Multiple testing adjusted *p*-value** | |
| --- | --- | --- |
| macromolecule metabolic process | 3.3E-4 |  |
| cellular metabolic process | 1.9E-4 |  |
| nitrogen compound metabolic process | 2.7E-3 |  |
| primary metabolic process | 7.0E-3 |  |
| ribonucleoprotein complex biogenesis | 1.1E-2 |  |
| regulation of metabolic process | 6.8E-2 |  |
| biosynthetic process | 1.7E-1 |  |
| positive regulation of metabolic process | 1.5E-1 |  |
| cell cycle process | 1.4E-1 |  |
| negative regulation of cellular process | 2.5E-1 |  |

**Supplementary Table 17:** Top 10 GO biological processes for constitutive USF1 target genes

| **Biological process** | **Multiple testing adjusted *p*-value** | |
| --- | --- | --- |
| cellular metabolic process | 1.2E-12 |  |
| primary metabolic process | 3.8E-11 |  |
| macromolecule metabolic process | 3.1E-8 |  |
| establishment of protein localization | 2.3E-5 |  |
| macromolecule localization | 2.2E-5 |  |
| nitrogen compound metabolic process | 4.2E-5 |  |
| catabolic process | 1.4E-4 |  |
| organelle organization | 1.3E-4 |  |
| establishment of localization in cell | 2.3E-4 |  |
| cellular localization | 2.3E-4 |  |

**Supplementary Table 18:** Top 10 GO biological processes for constitutive YY1 target genes

| **Biological process** | **Multiple testing adjusted *p*-value** | |
| --- | --- | --- |
| cellular metabolic process | 9.5E-101 |  |
| macromolecule metabolic process | 2.5E-78 |  |
| primary metabolic process | 9.0E-67 |  |
| nitrogen compound metabolic process | 1.3E-45 |  |
| biosynthetic process | 1.6E-34 |  |
| organelle organization | 7.0E-16 |  |
| cell cycle | 1.6E-15 |  |
| ribonucleoprotein complex biogenesis | 2.0E-14 |  |
| establishment of protein localization | 3.8E-14 |  |
| catabolic process | 2.4E-11 |  |

**Supplementary Table 19:** Top 10 GO biological processes for constitutive ZBTB33 target genes

| **Biological process** | **Multiple testing adjusted *p*-value** | |
| --- | --- | --- |
| cellular metabolic process | 8.5E-17 |  |
| macromolecule metabolic process | 6.0E-13 |  |
| nitrogen compound metabolic process | 7.4E-11 |  |
| primary metabolic process | 2.2E-10 |  |
| biosynthetic process | 3.1E-6 |  |
| ribonucleoprotein complex biogenesis | 1.7E-5 |  |
| cell cycle | 5.3E-5 |  |
| cell cycle process | 1.6E-4 |  |
| organelle organization | 6.3E-4 |  |
| cellular macromolecular complex subunit organization | 9.9E-4 |  |
