# Supplementary material for: T-KDE: a method for genome-wide identification of constitutive protein binding sites from multiple ChIP-seq data sets
Source: BMC Genomics. 2014 Jan 15;15:27. doi: 10.1186/1471-2164-15-27 (PMC3903014; doi:10.1186/1471-2164-15-27)
Supplement: Additional file 2 — Outlines of algorithms. T-KDE. [file 1471-2164-15-27-S2.docx]

| **Algorithm 1**: T-KDE |
| --- |
| *Input*: ChIP-seq peaks from multiple cell lines, and a user-defined $\theta$, the proportion of peak centers under a modal region required to declare it as a constitutive mode. |
| *Output*: a list of constitutive modes. |
| Perform the following steps for each chromosome:  1: Pool all ChIP-seq peak centers from all cell lines into a single data set.  2: Construct a binary range tree to partition the peak centers into terminal leaves. |
| 3: Traverse the binary range tree to obtain locations and location bound for data points in each leaf.  4: Density estimation with a KDE using all data points in each leaf.  5: Find the mode(s) of the density in each leaf.  6: Check if the proportion of peak centers under the modal region meets the threshold $\theta$ for a constitutive mode. |

| **Algorithm 2**: Recursive function for constructing a binary range tree containing constitutive clusters as terminal nodes. |
| --- |
| *Given*: a vector of ChIP-Seq peak centers, and a threshold $\theta$ (defined in algorithm 1) |
| *Output*: binary range tree data structure |
| 1: Calculate midrange location (mean of minimum and maximum) for the current list of ChIP-seq peak centers.  2: Split the list of peak centers at their midrange location, creating a left and a right partition.  3: If both partitions remain constitutive, repeat the process on each partition. |
| 4: Otherwise, ignore the split and stop growing the tree. |

| **Algorithm 3**: Identification of constitutive binding sites using MACS as suggested by a reviewer |
| --- |
| 1: For each BAM file, remove duplicate reads that map to exact same location.  2: Using MACS with default parameters to call peaks on the combined non-duplicate reads from all BAM files. |
| 3: For each peak compute the summary statistic of read count variability over a window ±50 bases from the MACS summit (var(log(read count+1))), declare loci with sufficiently low read count variability as constitutive. |
