# Supplementary material for: T-KDE: a method for genome-wide identification of constitutive protein binding sites from multiple ChIP-seq data sets
Source: BMC Genomics. 2014 Jan 15;15:27. doi: 10.1186/1471-2164-15-27 (PMC3903014; doi:10.1186/1471-2164-15-27)
Supplement: Additional file 3: Supplementary Figure S1 — Proportion of TKDE-declared versus KDE-declared constitutive CTCF binding sites whose distance from nearest motif-based constitutive CTCF binding site on 23 chromosomesare less than distance d plotted as a function of d for various bandwidths.Separate curves for T-KDE with bandwidth of 100 bps and for the same density estimation algorithm without the binary range tree pre-processing. [file 1471-2164-15-27-S3.docx]

**Figure S1:** Proportion of TKDE-declared versus KDE-declared constitutive CTCF binding sites whose distance from nearest motif-based constitutive CTCF binding site on 23 chromosomes are less than distance *d* plotted as a function of *d* for various bandwidths. Separate curves for T-KDE with bandwidth of 100 bps and for the same density estimation algorithm without the binary range tree pre-processing.

**
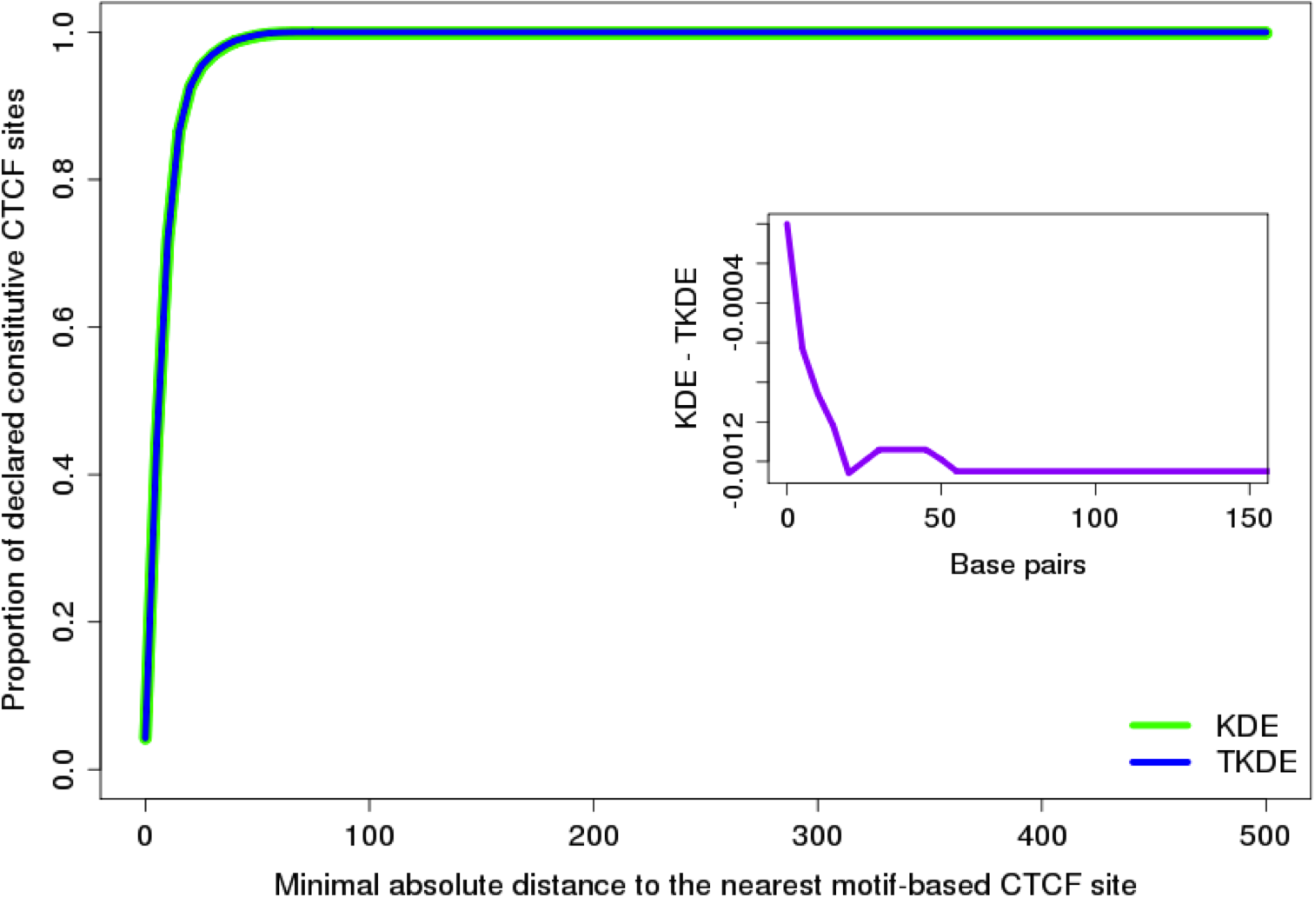
**
