# Supplementary material for: T-KDE: a method for genome-wide identification of constitutive protein binding sites from multiple ChIP-seq data sets
Source: BMC Genomics. 2014 Jan 15;15:27. doi: 10.1186/1471-2164-15-27 (PMC3903014; doi:10.1186/1471-2164-15-27)
Supplement: Additional file 4 — Additional results comparing various methods. T-KDE: A method for genome-wide identification of constitutive protein binding sites from multiple ChIP-seq data sets. [file 1471-2164-15-27-S4.docx]

Supplementary text for

T-KDE: A method for genome-wide identification of constitutive protein binding sites from multiple ChIP-seq data sets

Our paper proposes to apply kernel density estimation to the locations of peak centers as a way of detecting constitutive protein binding sites using ChIP-seq peaks from multiple cell lines. A reviewer suggested an alternative approach (algorithm S3 in additional file 2) that involved applying the peak-calling tool MACS to a concatenated data set combining reads from multiple ChIP-seq experiments. We have implemented the reviewer’s proposal and compared the results to T-KDE and to binning.

As mentioned in the Introduction, a drawback to using peak-calling algorithms to detect constitutive binding sites is the large volume of data involved. To compare the performance among T-KDE, the binning method, and the algorithm S3, we used CTCF data from only 12 cell lines to restrict the storage required. We applied the proposed algorithm S3 to the reads data in BAM format and applied both T-KDE and the binning method to the peak centers reported by ENCODE from the same cell lines.

In order to use MACS on multiple cell lines, we combined BAM files from different experiments into a single BAM file. We converted all BAM files to the SAM format, generated one header file, then converted the combined SAM files back to the BAM format. The size of the combined BAM file and the generated SAM file was ~40 GB whereas the size of the corresponding input files for T-KDE or binning were only ~18MB.

Next, we ran MACS on the combined data using default parameters and identified 89,592 CTCF binding sites in. By contrast, T-KDE identified 154,875 CTCF binding sites (32,100 declared constitutive) and binning identified 180,134 sites (27,783 declared constitutive). Notice that the total number of CTCF binding sites that MACS identified in the combined data was smaller than the number identified by either T-KDE or binning using peak centers reported by ENCODE from the same data. This discrepancy in total binding sites identified between MACS and T-KDE or binning seems consistent with our idea, expressed in the Introduction, that MACS may not be well suited for peak-finding when many separate ChIP-seq data sets from different cell lines are combined and processed simultaneously.

In order to compute the variance in log(read counts+1)) for all peak summits identified by MACS, we used the BAM file to get base-by-base alignments. This procedure is a computationally intensive. The histogram of the variation statistic is shown in Supplementary Figure S2. More than 98% of the peaks have a value < 1.

T-KDE identified 32,100 and binning identified 27,783 sites as constitutive. Lacking a defensible benchmark for a site’s having “sufficiently low” variance in log(read counts +1) to be declared constitutive, we followed the reviewer’s suggestion and declared the top-ranked 32,100 of MACS summits as constitutive, the same number as T-KDE declared. Recall the motif-based approach in the manuscript identified 17,575 constitutive sites using all 132 CTCF data sets. For T-KDE, for binning, and for the algorithm S3, we plotted the proportion of CTCF binding sites declared constitutive whose distance from the nearest motif-based constitutive CTCF binding site on all chromosomes was less than distance *d* as a function of *d* (Supplementary Figure S3). About 94% of the T-KDE-declared constitutive sites were within 50 bp of a motif-based constitutive site. For binning-declared sites, this percentage was lower at 25%, but rose to nearly 84% for binning-declared sites within 200 bp of motif-based constitutive sites. For the sites declared constitutive using the algorithm S3, the percentage of constitutive sites closer than 50 bp to a motif-based constitutive site at ~34% was slightly higher than binning but much lower than T-KDE. Expanding the neighborhood from within 50 bp to within 200 bp of a motif-based constitutive site, this value rose to only ~35% for the algorithm S3, much lower than the comparable values either for binning or for T-KDE.


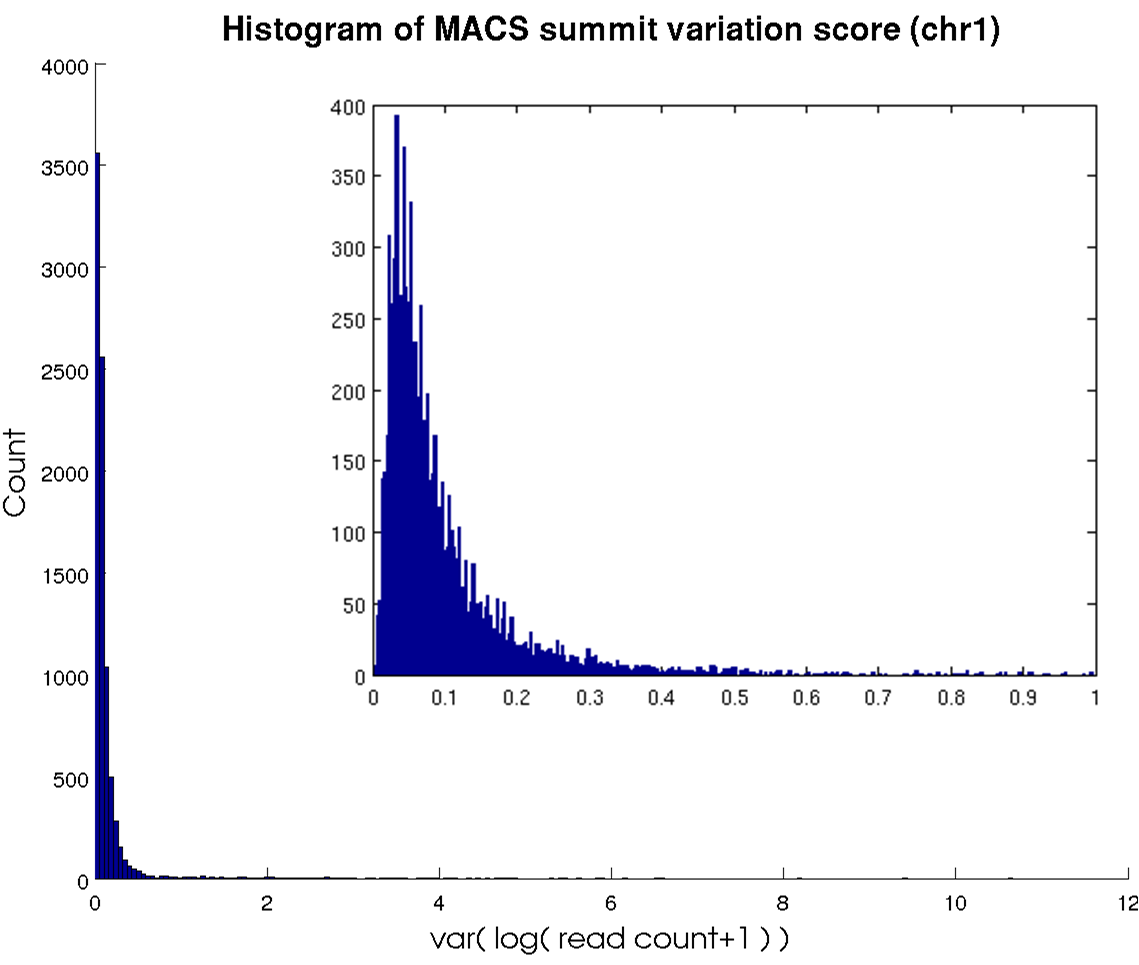


Supplementary Figure S2: Histograms of the variance of log(read count +1) within ±50 bp of a MACS summit for chromosome 1 (left bottom). The low-variance region is rescaled in the inset (top right).


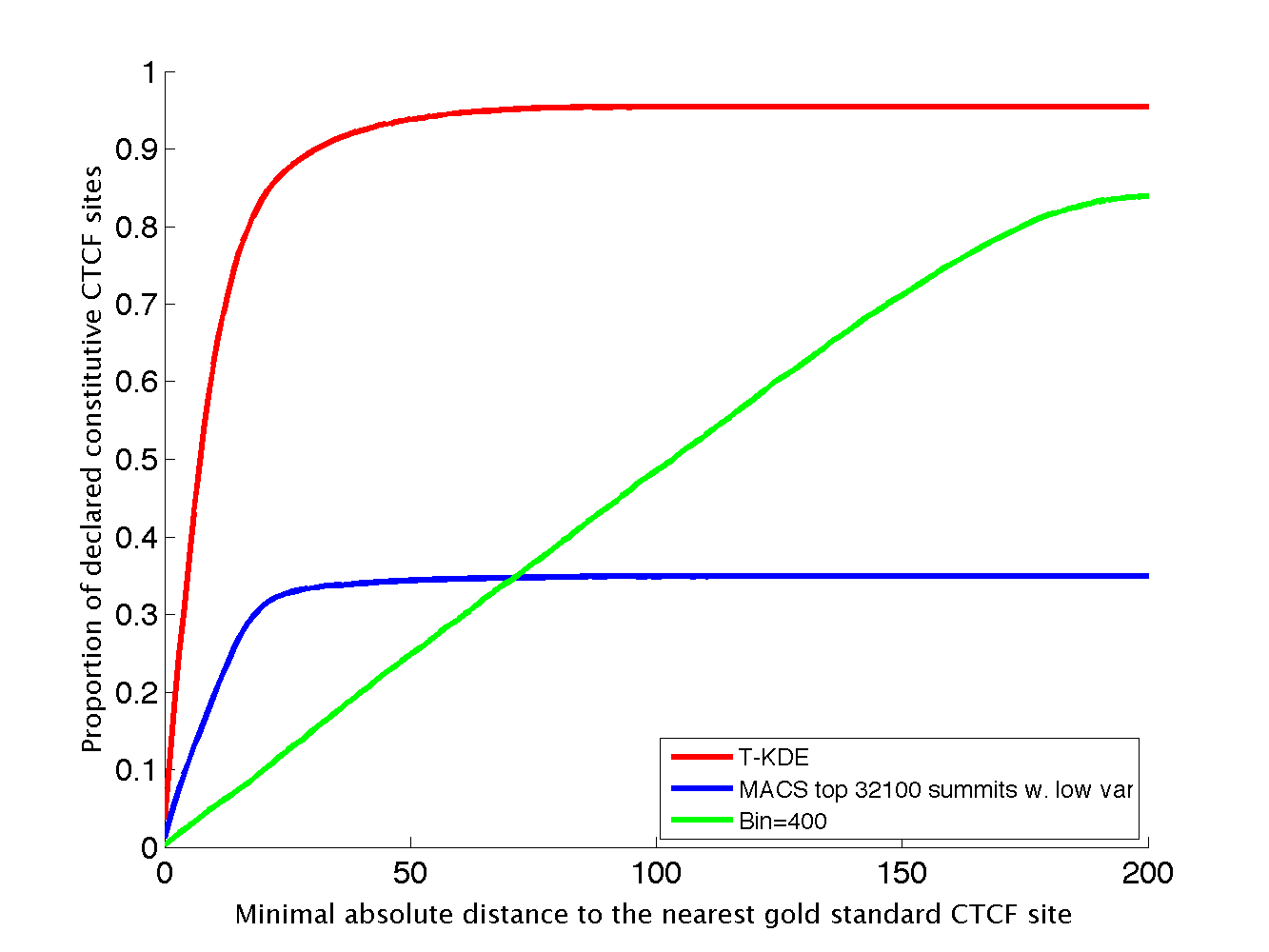


**Supplementary Figure S3**: Proportion of CTCF binding sites declared constitutive whose distance from the nearest motif-based constitutive CTCF binding site on all chromosomes was less than distance.
